# General Assessment of Indole Derivatives and Wnt Pathway as Potential Key Factors Against Various Diseases, with Particular Emphasis on Thyroid Tumors

**DOI:** 10.3390/ijms27167280

**Published:** 2026-08-14

**Authors:** Anna K. Skoczyńska, Andrzej Lewiński, Małgorzata Karbownik-Lewińska

**Affiliations:** 1 Department of Endocrinology and Metabolic Diseases, Medical University of Lodz, 281/289 Rzgowska St., 93-338 Lodz, Poland; anna.skoczynska@umed.lodz.pl; 2 Polish Mother’s Memorial Hospital—Research Institute, 281/289 Rzgowska St., 93-338 Lodz, Poland

**Keywords:** indoles, therapeutic activity, medicines, Wnt pathway, thyroid tumors

## Abstract

Indoles are chemical compounds that naturally occur in *Prokaryotes* and *Eukaryotes*. For example, indole alkaloids mainly occur in plant families such as *Nyssaceae, Rubiaceae, Apocynaceae,* and *Loganiaceae*. The popularity of indoles has led to further research into their function and synthetic modification. Meanwhile, the Wnt (Wingless and Int-1) signaling pathway is responsible for cell regulatory processes, while its dysfunction often constitutes the molecular basis for the pathogenesis of neoplastic processes. Databases such as PubMed, Scopus, Google Scholar, and Science Direct were used to perform a literature search with keywords including “indoles” and those related to their biological activity, as well as “Wnt pathway” and “thyroid cancer (TC)”. We used ClinicalTrials.gov to search current clinical trials for selected indoles, such as melatonin, indole-3-propionic acid, and indole-3-carbinol. While indoles possess many therapeutic properties, we focused here on the following: antitumor, antiviral, anti-inflammatory, antidepressant, antimigraine, antiemetic, and antihypertensive activities. We analyzed the Wnt pathway and its disruptions in thyroid cancer. In this manuscript, seventy-six (76) indoles, which have natural and synthetic origins, are presented. In addition to all activities mentioned, we discuss the therapeutic goals of indole applications in anticancer research. The Wnt signaling pathway can be changed in tumor cells, and such disruptions can occur in thyroid cancers.

## 1. Introduction

The Wnt (Wingless and Int-1) signaling pathway is responsible for cell regulatory processes, while its dysfunction often constitutes the molecular basis for carcinogenesis [1]. Cancer is a complex disease process that undoubtedly threatens human life, regardless of where it occurs in the body. Although cancer cells are characterized by uncontrolled growth, many other different mechanisms may be responsible for the development of this disease [2,3]. Epidemiological data from Eurostat suggest that cancer was the most common cause of death for people under 65 years old in the European Union in 2021 [4]. The World Health Organization (WHO) has revealed that cancer is the most common cause of death, ranking above ischemic disease and stroke [5]. Despite progress in cancer diagnostics and treatment, the success of anticancer therapy remains a challenge. There is a constant need to improve and develop new and safe anticancer medications [2,6,7]. This is due to the lack of selectivity of conventional chemotherapeutic agents and to multidrug resistance [2,8].

Apart from cancer, disturbances to the Wnt pathway have been observed in neurodegenerative and metabolic diseases [2,9,10]. There are two main pathway types: canonical (i.e., the Wnt/β-catenin pathway) and non-canonical (i.e., without the involvement of β-catenin). In addition, there is a non-canonical Wnt pathway that depends on the concentration of calcium ions. The Wnt/β-catenin pathway is a major signaling pathway that plays an important role in the epithelial–mesenchymal transition (EMT) because the target genes of this pathway are involved in cellular proliferation and differentiation. Disturbances of the Wnt/β-catenin pathway may lead to the development of thyroid cancer (TC). Therefore, drugs are being sought that affect proteins influencing this pathway.

Various histological types of TC can develop from thyroid follicular cells. The most frequently diagnosed are differentiated thyroid cancers (DTCs), of which approximately 80% are papillary thyroid cancers (PTCs), while follicular thyroid cancer (FTC) is less frequently diagnosed. Disturbances in the transmission of Wnt pathway signals may be the basis for oncogenesis in thyroid cells.

**Figure 1:**
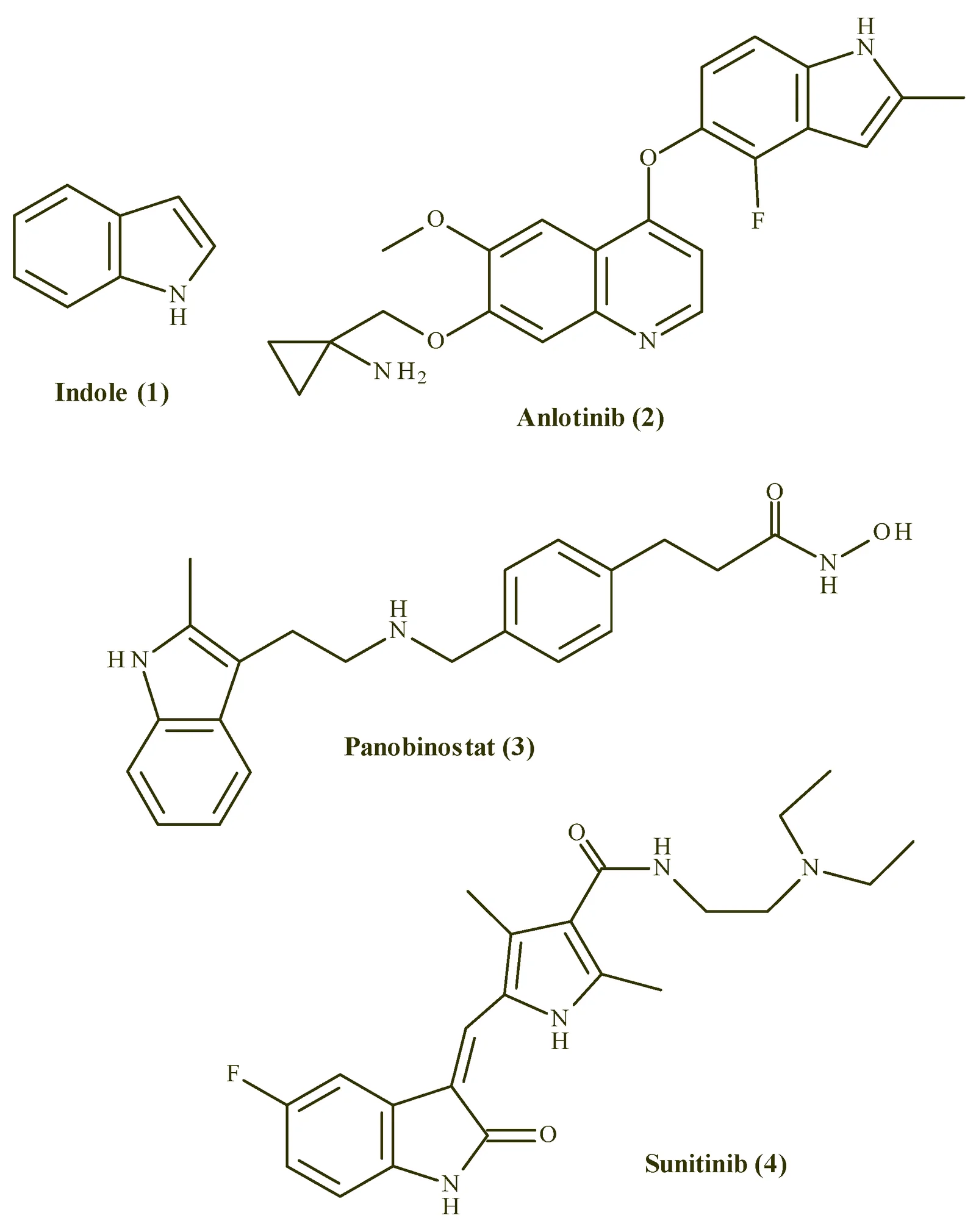
Structures **1**–**4**.

Indole (**1**) (Figure 1) and its derivatives show a wide spectrum of biological activity, which indicates the possibility of their use in medicine. In addition to their anticancer effects, they also have antiviral (including anti-HIV), anti-inflammatory, antioxidant, and antidiabetic properties. Confirmation of their medicinal properties lies in the fact that the Food and Drug Administration (FDA) has approved compounds for the treatment of cancer that contain an indole ring in their chemical structure, including anlotinib (**2**), panobinostat (**3**), and sunitinib (**4**) (Figure 1). The results of previously published experimental work indicate various biological properties of indole and its derivatives, which has led to great interest regarding this group of compounds. Due to the constant need to search for anticancer drugs, much attention is focused on chemical compounds that occur naturally, and the introduced chemical modifications of substituents often result in an increase in the cytotoxic activity of these compounds [2].

## 2. Indoles—A Short Description of Their Characteristics and Medicinal Properties

**Figure 2:**
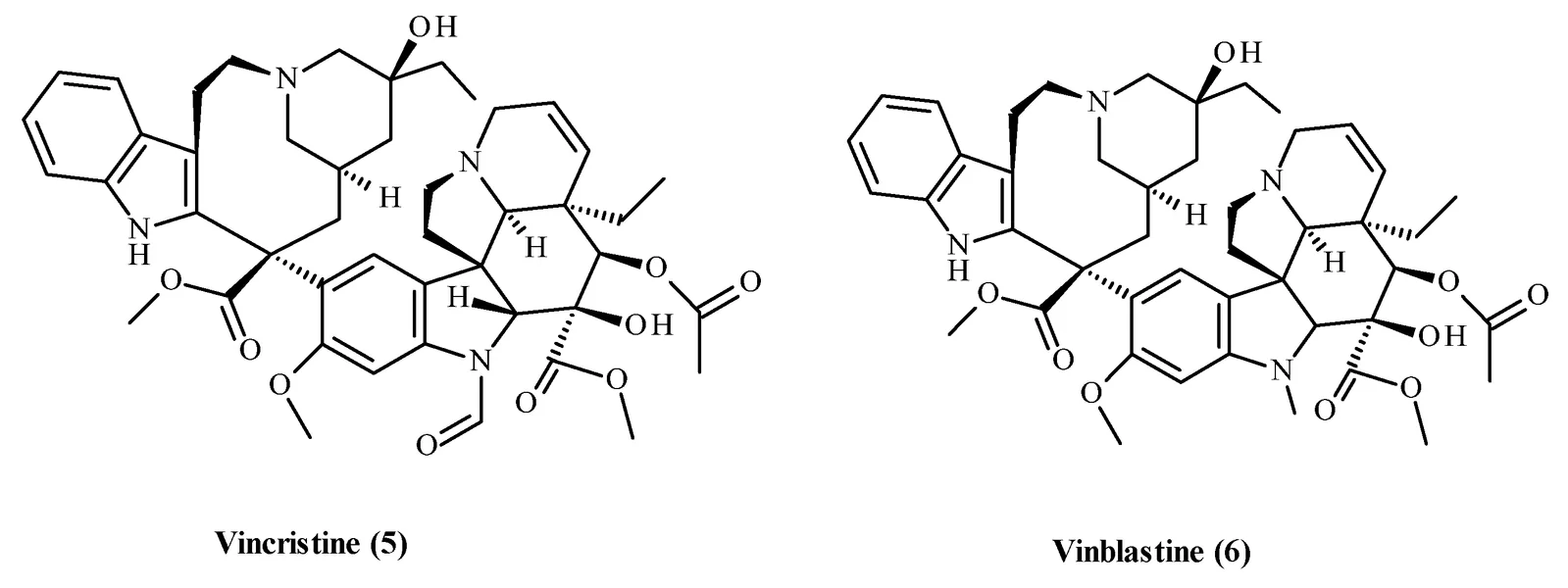
Structures of vincristine (**5**) and vinblastine (**6**).

The indole ring is one of the most common heterocyclic systems in bioactive organic compounds of natural and synthetic origin. It is an element of the structure of drugs used in cancer chemotherapy. Indole and its derivatives occur in natural products in the form of alkaloids, as well as in plant and animal hormones [2,11,12]. They have many pharmacological properties, such as antibacterial, antifungal [13,14,15,16,17,18], anti-inflammatory [13,19,20,21,22,23], antihistamine [13,24,25], antioxidant [13,26,27,28,29], antidiabetic [13,30], antiviral [13,31], anticholinergic [13,32,33], and anticancer [13,34,35,36,37,38,39]. Among naturally occurring indoles, the antimitotic effects of vincristine (**5**) and vinblastine (**6**) (Figure 2), which were isolated from *Catharanthus roseus*, have been used in the treatment of Hodgkin’s lymphoma, non-Hodgkin’s lymphoma, Kaposi’s sarcoma, and breast cancer [2,13,40].

**Figure 3:**
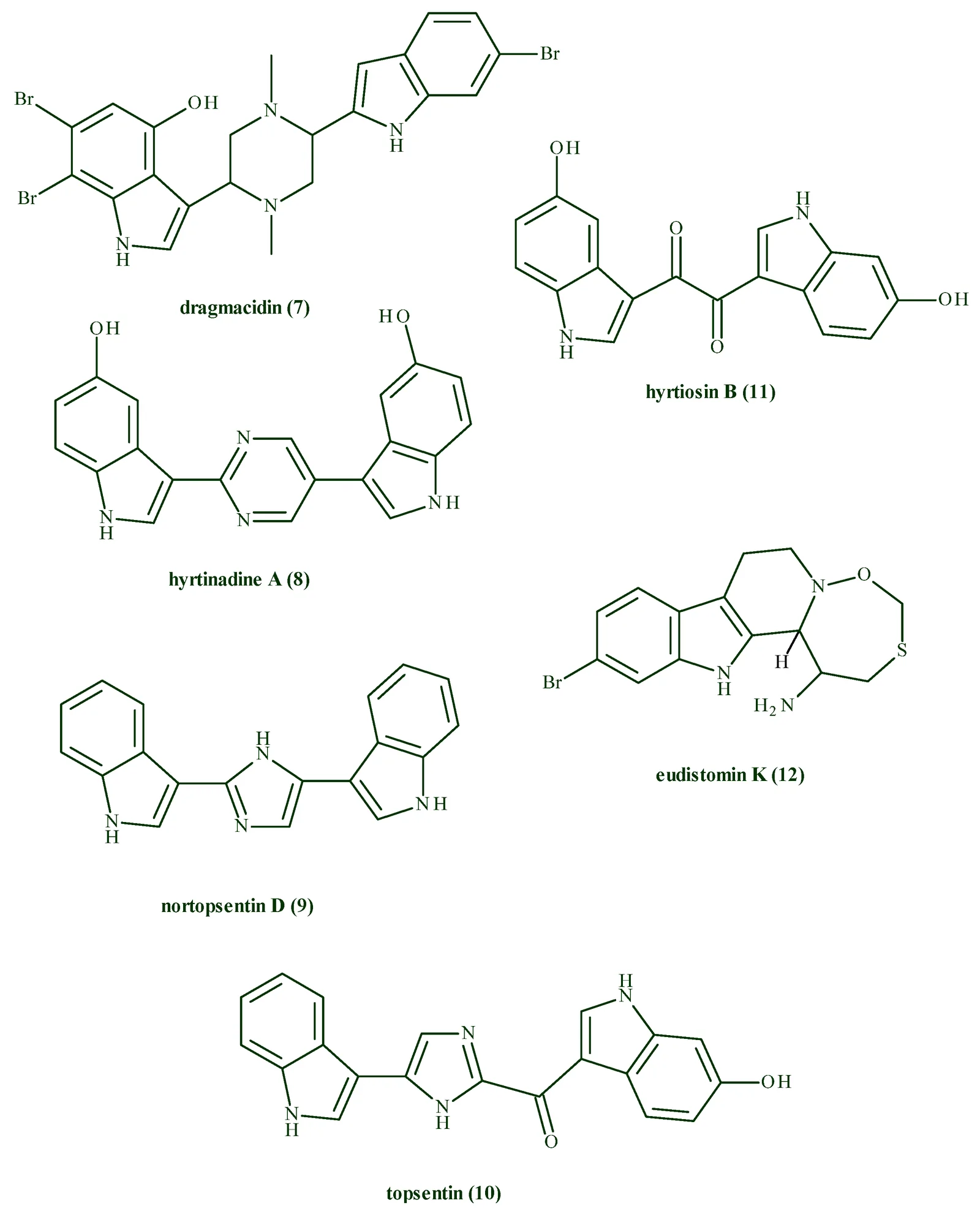
Structures **7**–**10**.

Another example of an anticancer indole derivative is mitomycin C, which is an antibiotic. It is activated by reduction, and then its neutral form is created, which forms extra- and intrastrand crosslinks with DNA in vivo [2,41]. Moreover, alkaloids composed of two indole rings, such as dragmacidin (**7**), hyrtinadine A (**8**), nortopsentin D (**9**), topsentin (**10**), and hyrtiosin B (**11**) (Figure 3), have shown cytotoxic activity against various cancer cell lines [13,42]. Eudistomin K (**12**) (Figure 3), which is a marine alkaloid, was shown to have antiproliferative activity against the P-388 cancer cell line. This indole derivative is considered a leading compound in the design of anticancer medications [13,43,44,45].

**Figure 4:**
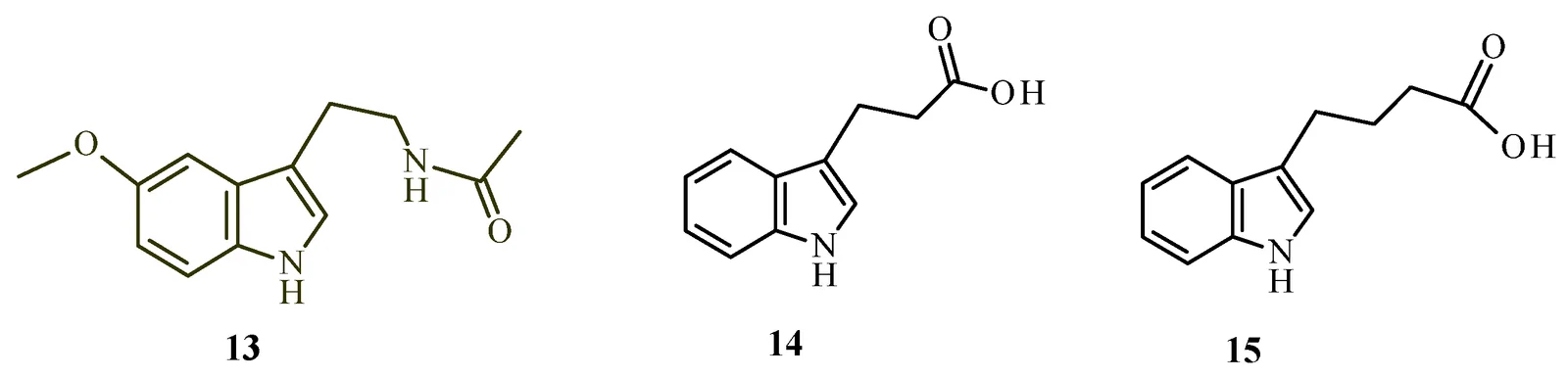
Melatonin (**13**), indole-3-propionic acid (**14**) and indole-3-butyric acid (**15**).

Melatonin (N-acetyl-5-methoxytryptamine) (**13**) (Figure 4) is an indole derivative that is secreted mainly by the pineal gland in animals. It is also present in smaller amounts in the eyes, lymphocytes, bone marrow, gastrointestinal tract, and the skin of humans and rodents [46]. Melatonin regulates circadian rhythms and influences sleep. It also occurs naturally in various fruits, including grapes, cherries, strawberries, apples, pineapples, pomegranates, and kiwifruit [47]. Among vegetables, the highest levels have been reported in tomatoes and peppers. Among legume seeds, the highest melatonin content has been found in white mustard (189 ng/g) and black mustard [47]. For many years, considerable attention has been devoted to **13** because of its protective and antioxidant properties, particularly in experimental studies of lipid peroxidation in the thyroid gland. Various in vitro experimental models have been used to investigate the protective effects of melatonin in porcine thyroid tissue. The results indicate that **13** does not increase oxidative damage to membrane lipids, as assessed by lipid peroxidation (LPO), in the thyroid beyond the physiological threshold. Studies using porcine thyroid tissue have demonstrated that melatonin exerts preventive effects by protecting membrane lipids against pro-oxidants, including Fe^2+^ ions [48], and sodium/iodide symporter inhibitors, such as ammonium thiocyanate (NH_4_SCN), potassium selenocyanate (KSeCN), and sodium fluoride (NaF) [49], which induce membrane lipid peroxidation in thyroid homogenates. It is worth noting that thyroid follicular cells and C cells possess genes encoding enzymes involved in melatonin biosynthesis, including acetylserotonin O-methyltransferase (ASMT) and aralkylamine N-acetyltransferase (AANAT) [50]. In addition, the melatonin receptor MT1 is also expressed in these cells [50]. Melatonin influences thyroid function and thyroid hormone concentrations in the blood. As an antioxidant, melatonin (**13**) scavenges free radicals and reactive species, including hydroxyl (•OH), alkoxy (RO•), peroxy (ROO•), and nitric oxide (•NO) radicals. Melatonin can also stimulate antioxidant enzymes, such as superoxide dismutase (SOD), catalase (CAT), glutathione reductase (GR), and glutathione peroxidase (GPx) [51,52,53]. In contrast, indole-3-propionic acid (**14**), which is structurally similar to melatonin (**13**), inhibits xanthine oxidase and nitric oxide synthase, which are pro-oxidant enzymes [46]. Melatonin (**13**) was the first documented antioxidant shown to protect against oxidative damage to membrane lipids caused by the Fenton reaction in the thyroid gland [48]. An experiment conducted in albino Wistar rats administered 2–4 mg/kg body weight of mercury chloride demonstrated that co-administration with **13** provided partial protection against oxidative damage induced by this inorganic salt [54]. In turn, treatment with nitrobenzene increased LPO levels in porcine thyroid homogenates, whereas melatonin prevented the damage induced by this synthetic chemical [55]. Another study using potassium bromate (KBrO_3_) showed that compounds **13** and **14** decreased LPO levels in the thyroid glands of rats injected with this potassium salt. However, this effect was not observed in vitro in experiments using porcine thyroid homogenates [56]. Numerous clinical trials involving compound **13** have been conducted. According to the ClinicalTrials.gov database, examples of such studies include investigations of uveal melanoma (melatonin as an adjuvant), childhood pneumonia (the role of melatonin as an adjuvant therapy), the effect of preoperative melatonin administration on recovery quality after elective laparoscopic cholecystectomy, and the association between melatonin and the risk of cardiovascular events and malignant tumors in older adults [57]. Compound **14** is also being investigated in clinical trials, including studies on multiple sclerosis and a pilot study evaluating its effects as a dietary supplement on the regulation of T cells and brain-derived neurotrophic factor (BDNF), as well as its potential effects on blood biomarkers used to assess the risk of metabolic disorders such as type 2 diabetes and cardiovascular disease [57].

**Figure 5:**
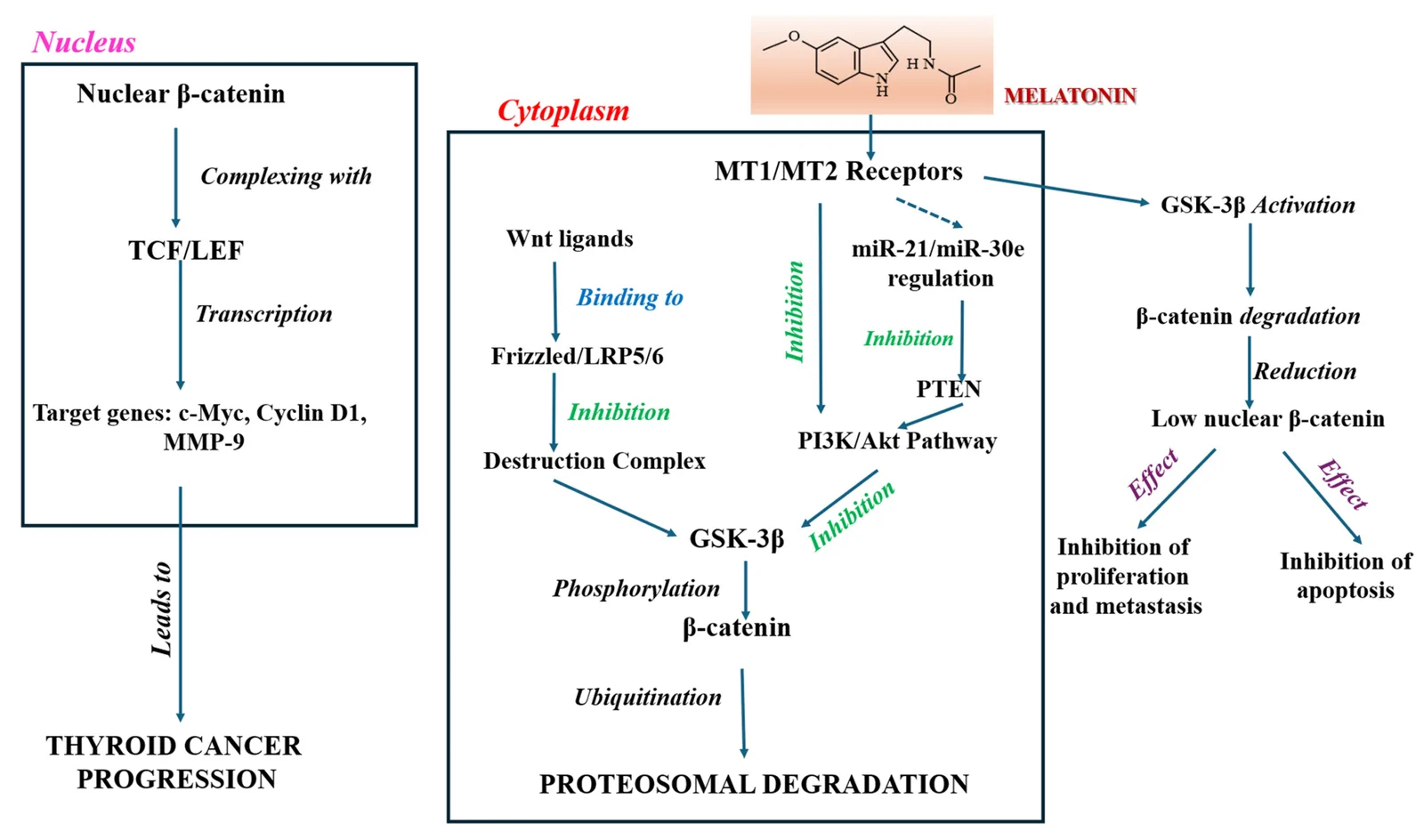
Proposed scheme illustrating the interaction of indole derivative with the Wnt signaling pathway in TC cells, using melatonin (**13**) as an example. (MT1/MT2 receptors—melatonin receptors).

In an experiment described by Cheng et al. [58], compound **13** affected the Wnt signaling pathway by downregulating the expression of Wnt5a and β-catenin in human gastric cancer cells (SGC-7901). In another study conducted on nasopharyngeal carcinoma cells, melatonin inhibited the Wnt/β-catenin signaling pathway by suppressing the nuclear translocation of β-catenin [59]. The antitumor activity of **13** was also investigated in papillary and anaplastic thyroid cancer cell lines. The results demonstrated that **13** inhibited the proliferation of the thyroid cancer cell lines tested; however, these effects were not observed at physiological concentrations (0.01–3 nM). Moreover, simultaneous irradiation and treatment with **13** increased the radiosensitivity of these thyroid cancer cells through inhibition of NF-κB/p65 phosphorylation [60]. Figure 5 presents a proposed scheme illustrating the interaction of an indole derivative with the Wnt signaling pathway in thyroid cancer cells, using melatonin as an example. This process represents a multistep inhibitory cascade in which melatonin (**13**) acts as a natural antagonist of the Wnt/β-catenin pathway. The process can be divided into three cellular compartments: the extracellular space, the cytoplasm, and the nucleus. Binding of melatonin (**13**) to its receptors, MT1/MT2, initiates intracellular signaling that promotes activation of the β-catenin destruction complex, in which GSK-3β plays a key role. Subsequent degradation of β-catenin prevents the proliferation and dissemination of cancer cells.

In turn, indole-3-butyric acid (**15**) (Figure 4) is a well-known plant growth hormone (auxin) that occurs naturally in *Arabidopsis thaliana*. This indole derivative was also experimentally investigated as a potential antioxidant in porcine thyroid homogenates. It was shown that **15** exhibited promising protective activity against oxidative damage induced by the Fenton reaction (Fe^2+^ + H_2_O_2_ → Fe^3+^ + •OH + OH^−^), as it decreased experimentally induced lipid peroxidation (LPO). Therefore, **15** may be considered a potential dietary supplement because of its protective effects on thyroid membrane lipids [61]. Interesting results were obtained when KIO_3_ was used as a pro-oxidant in homogenates of different porcine tissues (thyroid, ovary, spleen, liver, brain, intestine, and kidney), with melatonin used as a potential antioxidant. Compound **13** was more effective at higher KIO_3_ concentrations, and the strongest antioxidant effect of melatonin was observed in thyroid homogenates [62]. Of particular importance was the observation that compounds **13** and **14** exhibited a cumulative protective effect against KIO_3_-induced damage in porcine thyroid homogenates, as assessed by the lipid peroxidation assay [63]. Another study conducted on porcine skin homogenates revealed that compounds **13** and **14** (Figure 4) exerted protective effects against oxidative damage induced by high Fe^2+^ concentrations [64]. Further studies of lipid peroxidation in porcine thyroid and liver homogenates demonstrated that caffeic acid phenethyl ester (CAPE) decreased the level of LPO products induced by the Fenton reaction. Compound **13** exhibited a similar protective effect when used at the same concentrations [65]. Based on the results of an experiment conducted on hamster kidney tissue, compounds **13** and **14**, as well as ascorbic acid, were identified as promising agents for combination therapy with estrogens and may have potential pharmaceutical applications. A comparison of the antioxidant activities of **13** and 17β-estradiol against KIO_3_-induced lipid peroxidation in porcine thyroid homogenates revealed that melatonin was a more effective protective agent than 17β-estradiol [66]. Another interesting biological activity of **13** is its potential to accelerate tissue repair. Melatonin was shown to exert a regulatory effect on collagen accumulation in the scar tissue of infarcted hearts [67].

In addition to their anticancer properties, indole compounds exhibit a broad range of pharmacological activities with potential applications in drug development, including antiviral, anti-inflammatory, antidepressant, antimigraine, antiemetic, and antihypertensive activities. These represent only a selection of the numerous biological properties of indoles discussed in this paper.

**Figure 6:**
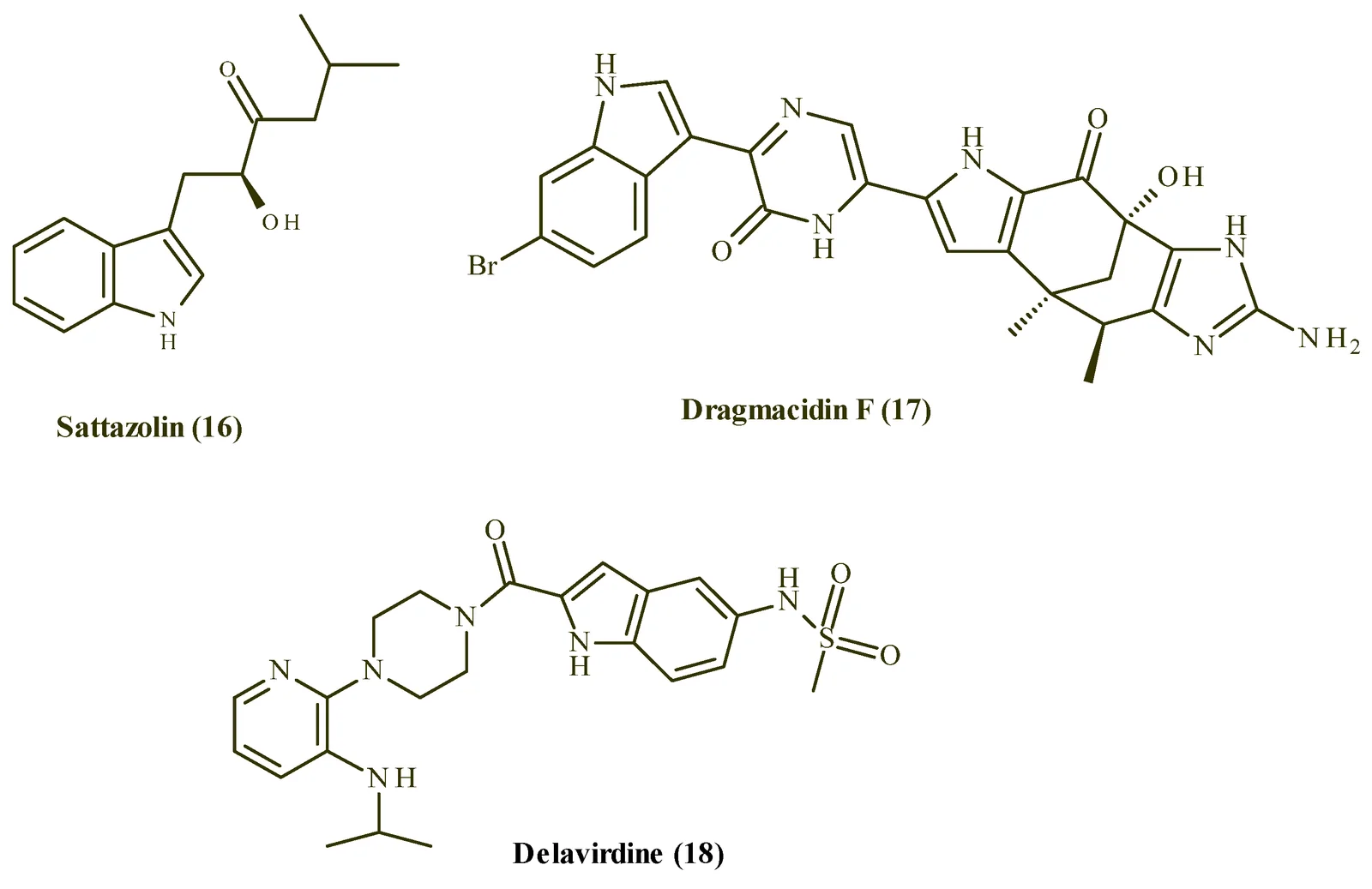
Structures **16**–**18**.

Viral diseases are widespread globally and include infections caused by influenza viruses, human immunodeficiency virus (HIV), hepatitis viruses, and Ebola virus. The indole derivative sattazolin (**16**) (Figure 6) is an indole acyloin that has demonstrated potential antiviral activity against HSV-1 and HSV-2 (*Herpes simplex virus*) [13,68]. Chen and co-workers reported the isolation of 17 new indole alkaloids and 14 known analogs from an aqueous extract of *Isatis indigotica* roots, which exhibited antiviral activity against influenza A virus A/Hanfang/359/95 (H3N2) [13,69]. Dragmacidins, isolated from marine sponges of the genus *Halicortex*, have demonstrated moderate antiviral activity. In particular, dragmacidin F (**17**) (Figure 6) exhibited activity against HSV-1 and HIV-1 [13,70]. Delavirdine (**18**) (Figure 6) is an antiretroviral agent approved by the FDA in 1997. It is a non-nucleoside reverse transcriptase inhibitor (NNRTI) used in the treatment of human immunodeficiency virus (HIV) infection. Delavirdine also inhibits the cytochrome P450 enzyme CYP3A4 and therefore has the potential to interact with numerous drugs. However, its clinical use is limited by its hepatotoxicity [13,71,72]. Based on the crystal structure of the delavirdine–HIV-1 reverse transcriptase complex, it has been proposed that the carbonyl and amino groups of the indole moiety form hydrogen bonds with lysine residues [13,73].

**Figure 7:**
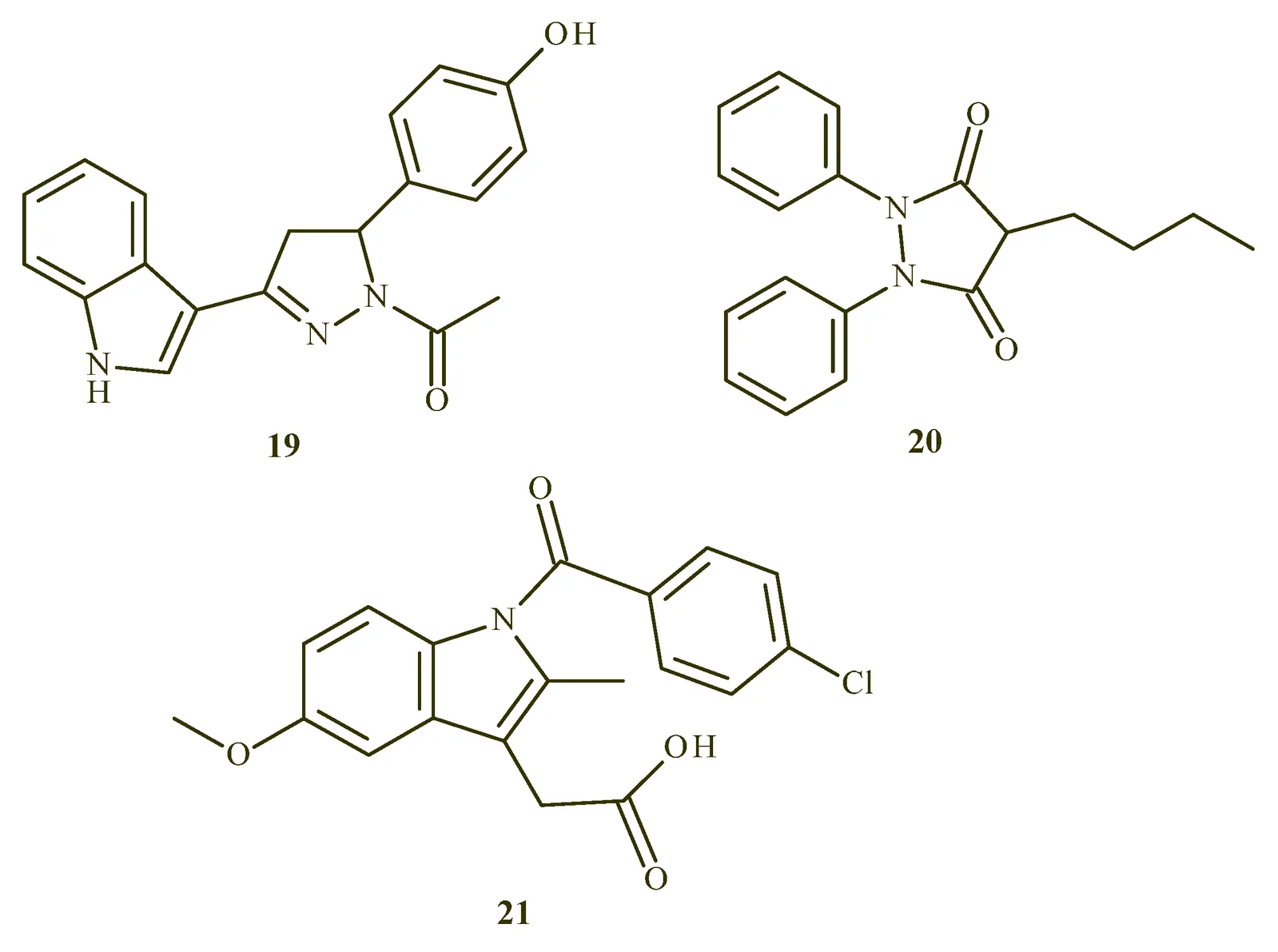
Structures **19**–**21**.

Several synthetic indole derivatives have been evaluated for their anti-inflammatory properties. Rani et al. synthesized and evaluated a series of indole derivatives based on chalcone and pyrazoline scaffolds. Among the compounds investigated, 3-[1-acetyl-5-(p-hydroxyphenyl)-2-pyrazolin-3-yl]indole (**19**) (Figure 7) exhibited the most promising anti-inflammatory activity, showing the greatest inhibition of edema, lower ulcerogenicity, and lower acute toxicity than the reference drug phenylbutazone (**20**) (Figure 7). However, its protective effect was weaker than that of indomethacin (**21**) (Figure 7) [13,74].

**Figure 8:**
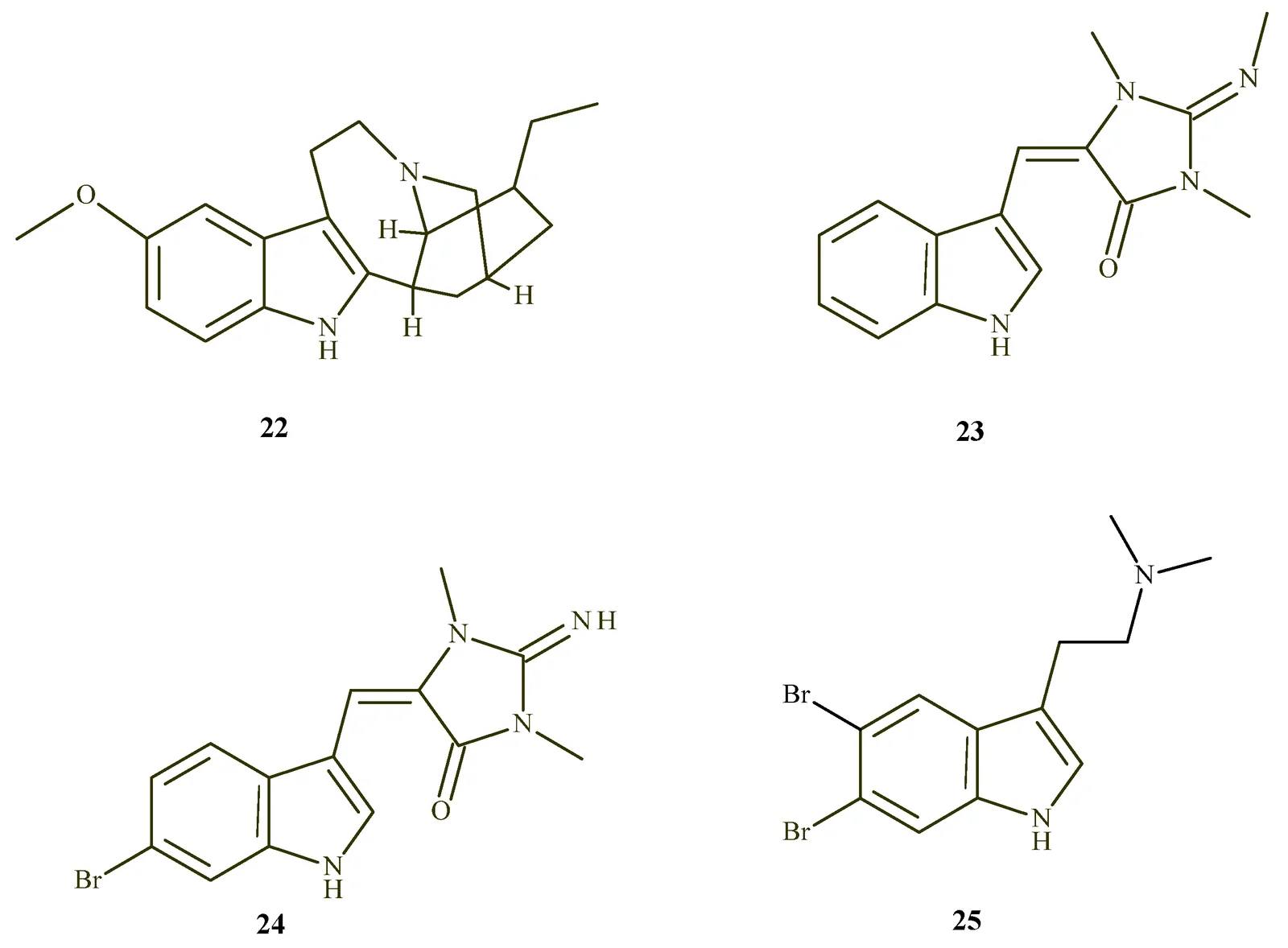
Structures **22**–**25**.

Singh et al. synthesized a series of compounds containing a bis-indole moiety and evaluated their anti-inflammatory activity. The most active compound in the series exhibited greater edema-inhibitory activity, as well as lower ulcerogenic potential and acute toxicity, than **20** (Figure 7) [13,75]. Antidepressants are a group of drugs used in the treatment of depressive disorders. *Tabernanthe iboga*, a tropical shrub native to Central Africa, exhibits central nervous system stimulant properties, which are primarily attributed to the presence of indole alkaloids. One representative compound is ibogaine (**22**) (Figure 8) [13,76]. Ibogaine has been shown to inhibit serotonin and dopamine transporters, providing a potential mechanistic basis for its psychoactive effects. Several marine-derived indole compounds have also demonstrated antidepressant-like or anxiolytic activity. Methylaplysinopsin (**23**) (Figure 8), isolated from *Aplysinopsis reticulata*, has been reported to interact with serotonergic mechanisms and to inhibit monoamine oxidase [13,77]. 6- 6-Bromoaplysinopsin (**24**) (Figure 8), isolated from *Smenospongia aurea*, exhibits affinity for the 5-HT2A and 5-HT2C receptors [13,78]. 5,6-Dibromo-N,N-dimethyltryptamine (**25**) (Figure 8) exhibited significant antidepressant-like activity in behavioral studies conducted in mice [13,77].

**Figure 9:**
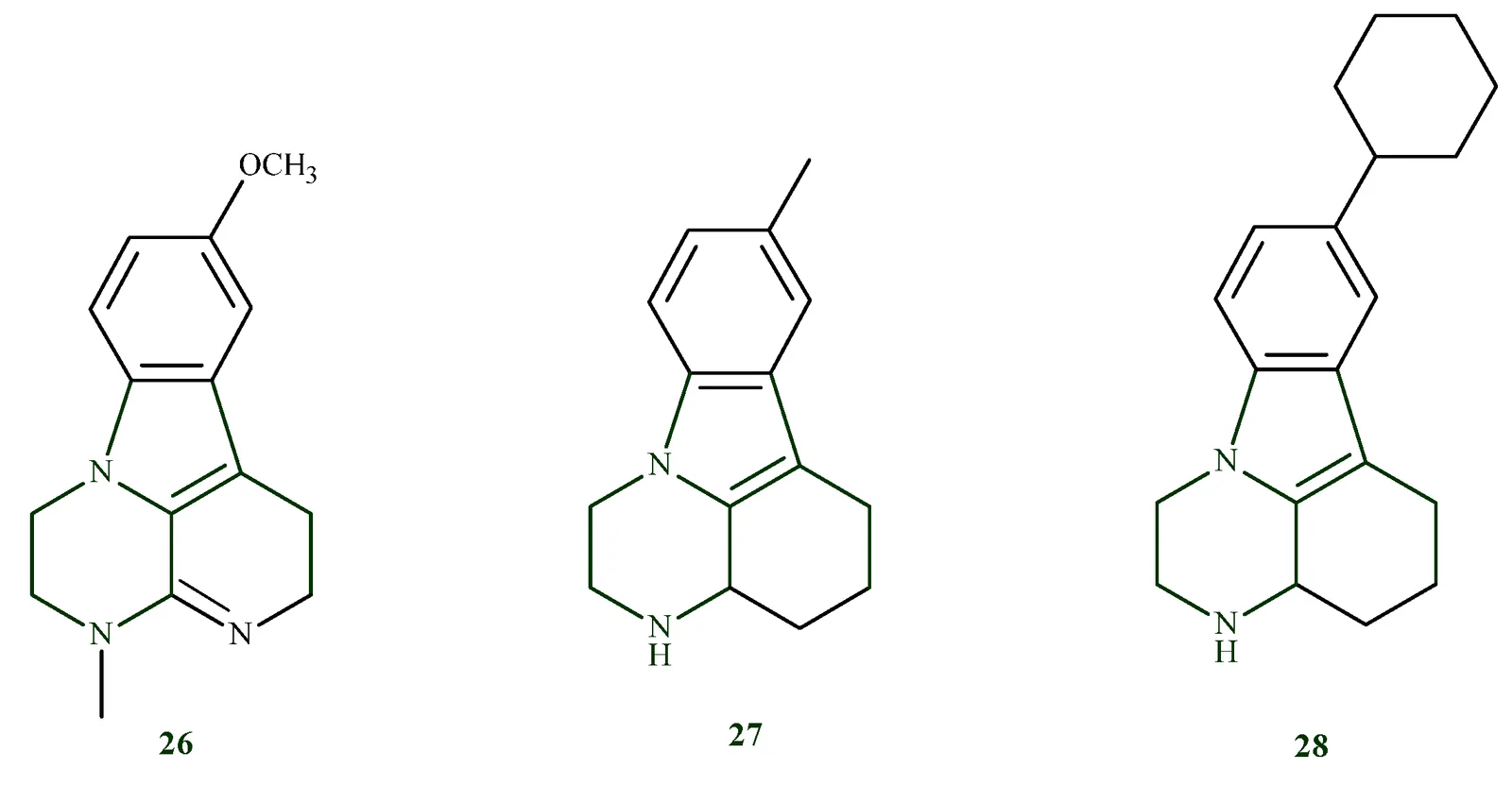
Structures **26**–**28**.

Some synthetic antidepressants developed in the 1990s act as reversible inhibitors of monoamine oxidase A (MAO-A). MAO-A catalyzes the oxidative deamination of monoamine neurotransmitters, including serotonin, dopamine, and norepinephrine. Inhibition of this enzyme prevents the degradation of these neurotransmitters and consequently increases their availability in the synaptic cleft. Three drugs, namely metralindole (**26**), pirlindole (**27**), and terindole (**28**) (Figure 9), are reversible inhibitors of MAO-A [13,79,80]. These compounds belong to the class of tetracyclic antidepressants and act as reversible monoamine oxidase inhibitors (RIMAs). Metralindole (**26**) is a triazolo-containing tetracyclic compound with a methoxy substituent, whereas pirlindole (**27**) and terindole (**28**) are structurally related tetracyclic derivatives bearing different substituents on the aromatic ring.

**Figure 10:**
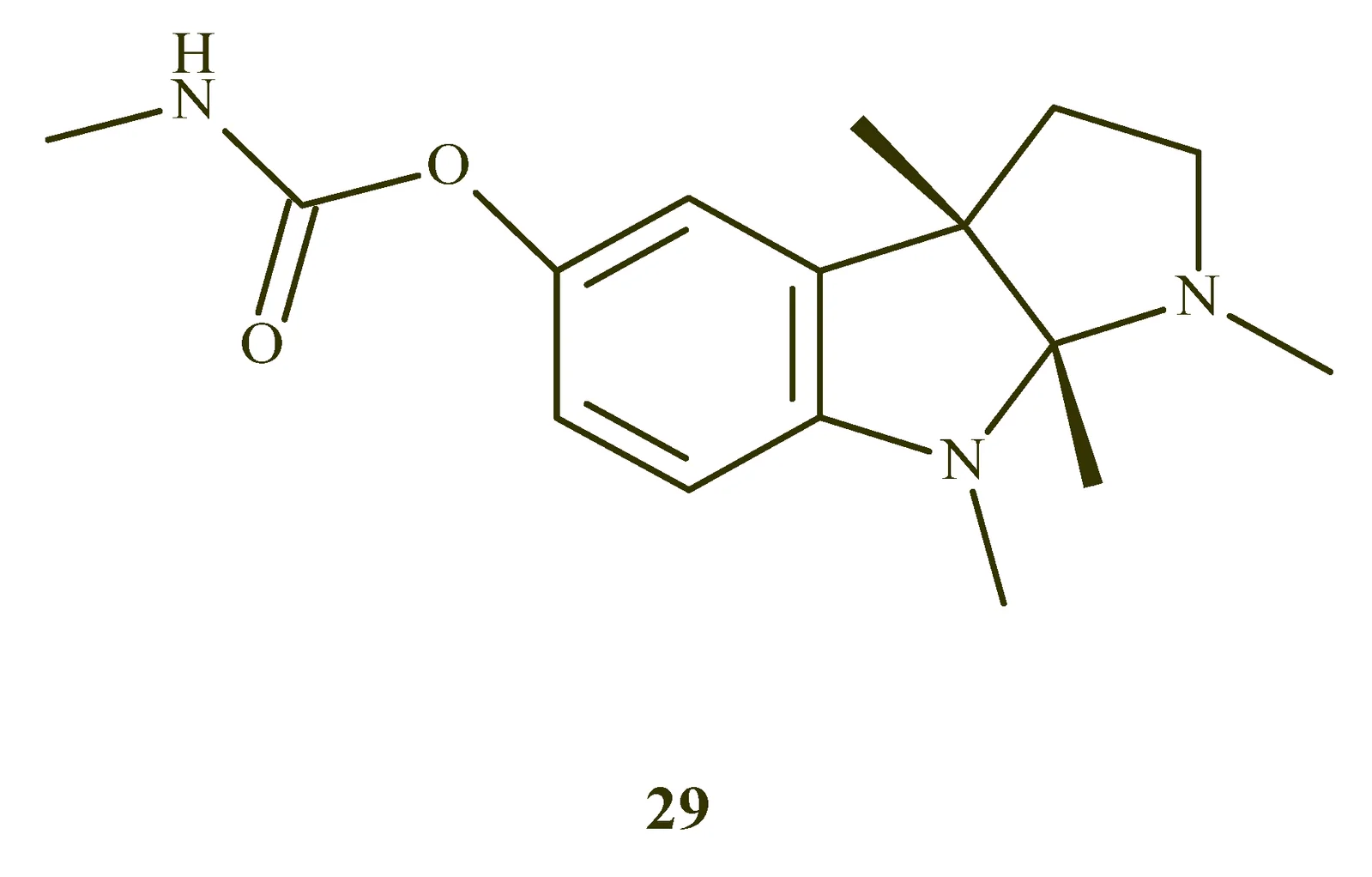
Structure of **29**.

Physostigmine (**29**) (Figure 10) is an important reversible acetylcholinesterase inhibitor derived from the Calabar bean (*Physostigma venenosum*). By inhibiting acetylcholinesterase, physostigmine increases the concentration of acetylcholine at cholinergic synapses and enhances cholinergic neurotransmission. Acetylcholinesterase inhibitors are used in the treatment of several disorders, including glaucoma, Alzheimer’s disease, delayed gastric emptying, and myasthenia gravis. Physostigmine has also been reported to improve memory and has been used in the treatment of anticholinergic, myasthenia gravis and orthostatic hypotension [13,81].

**Figure 11:**
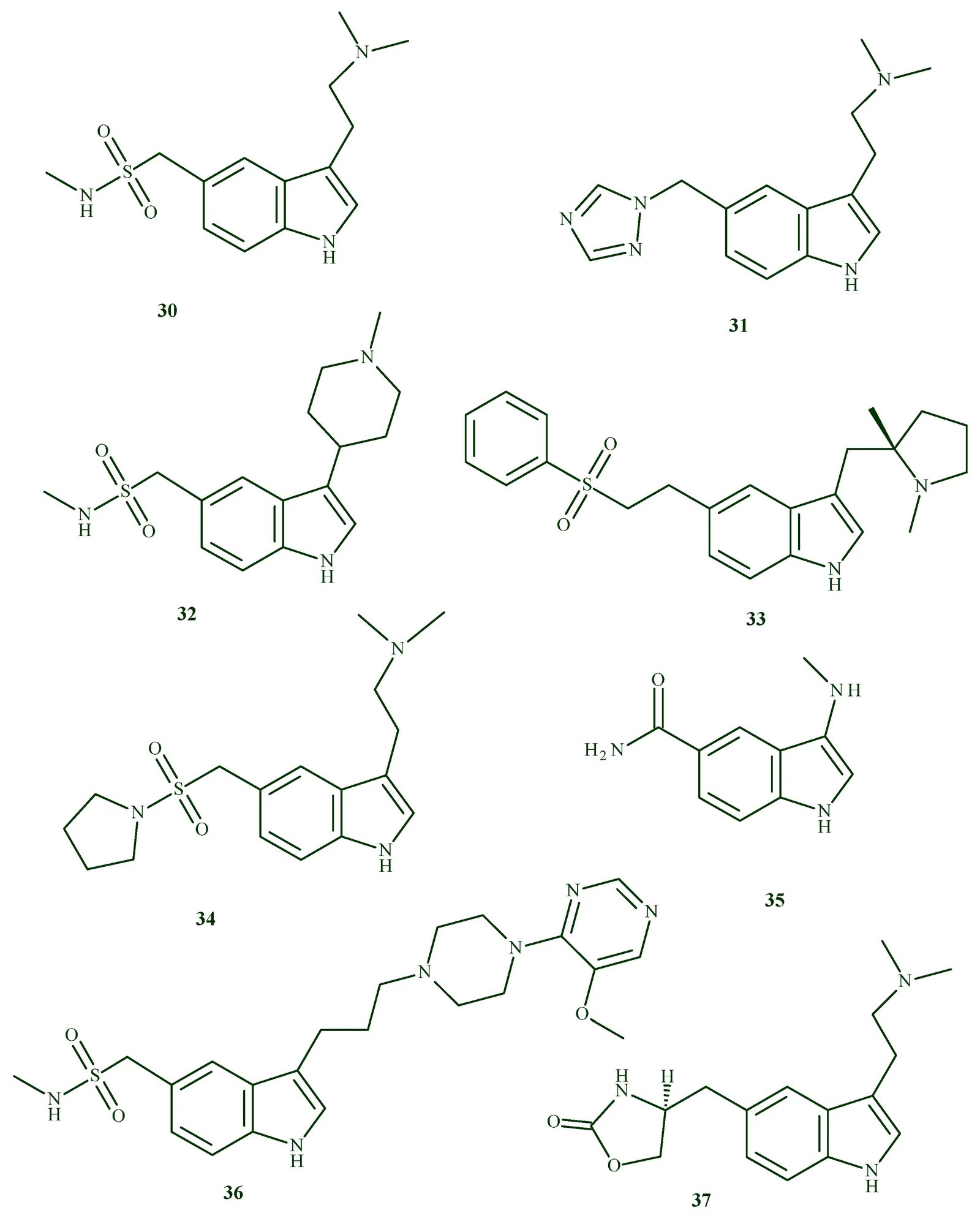
Structures **30**–**37**.

Migraine is a common neurological disorder characterized by recurrent, often unilateral, throbbing headache. It is estimated that migraine affects approximately 12–15% of the population [82]. Triptans are a class of tryptamine-based drugs commonly used for the acute treatment of migraine and include sumatriptan (**30**), rizatriptan (**31**), naratriptan (**32**), eletriptan (**33**), almotriptan (**34**), frovatriptan (**35**), avitriptan (**36**), and zolmitriptan (**37**) (Figure 11). These drugs exert their therapeutic effects primarily by acting as agonists at the 5-HT1B and 5-HT1D receptors, which are G protein-coupled receptors [13].

**Figure 12:**
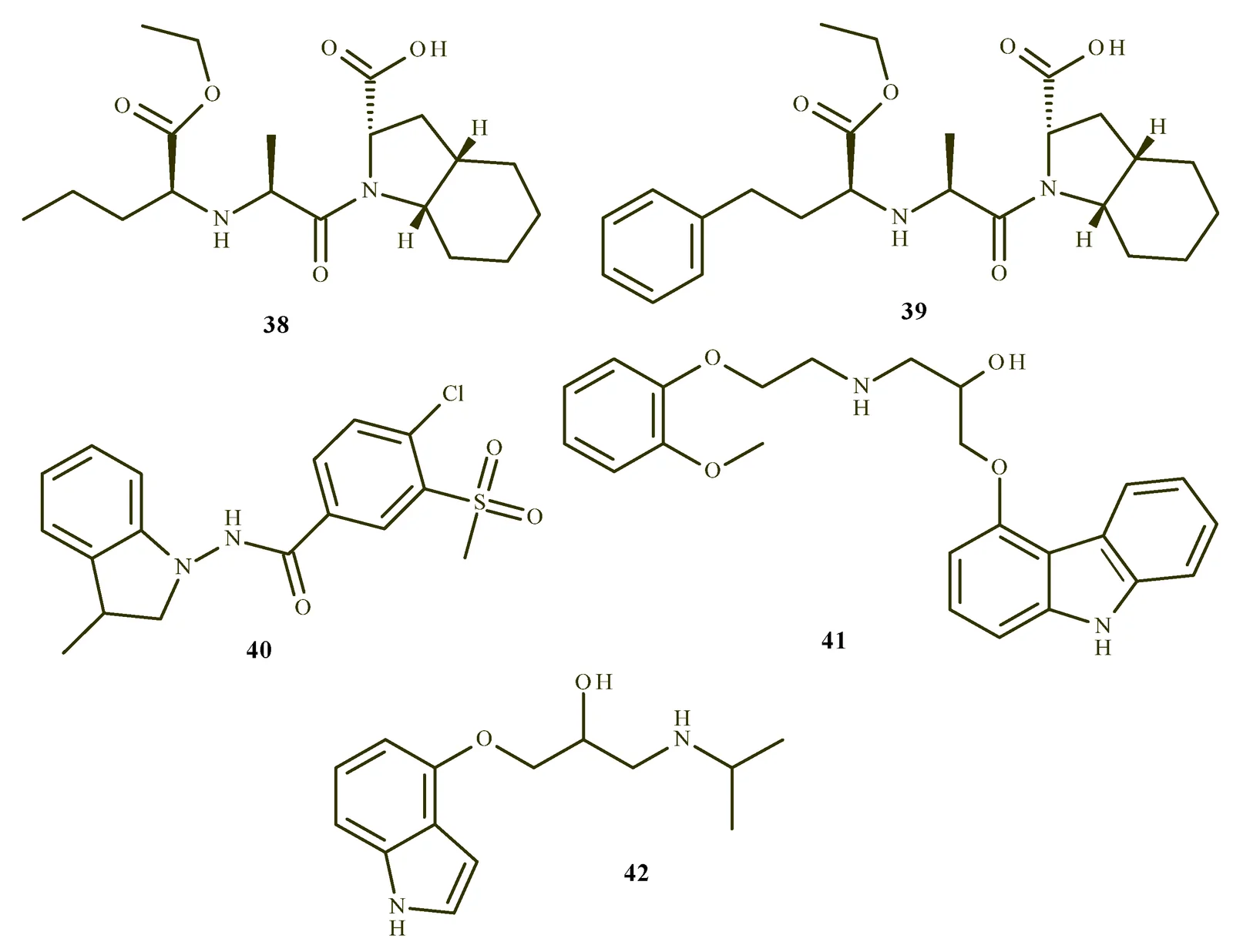
Structures **38**–**42**.

Indole-containing antihypertensive drugs exhibit diverse mechanisms of action and include α/β-blockers, angiotensin-converting enzyme (ACE) inhibitors, thiazide-like diuretics, and angiotensin II receptor type 1 (AT1) antagonists. Perindopril (**38**) and trandolapril (**39**) (Figure 12) are commercially available ACE inhibitors containing an octahydroindole moiety in their structures. They exert their antihypertensive effects by inhibiting the conversion of angiotensin I to angiotensin II, a key component of the renin–angiotensin–aldosterone system [13,83,84,85]. Adverse effects associated with these drugs include hypotension, dry cough, headache, dizziness, fatigue, nausea, and renal dysfunction. Indapamide (**40**) (Figure 12) is a dihydroindole-based drug belonging to the class of thiazide-like diuretics and is used in the treatment of hypertension and heart failure [13,84,86]. Common adverse effects include fatigue, orthostatic hypotension, allergic reactions, and hypokalemia. Carvedilol (**41**) (Figure 12) is a well-known β-adrenergic receptor blocker used in the treatment of congestive heart failure and hypertension. Pindolol (**42**) (Figure 12) is another β-adrenergic receptor blocker that received FDA approval in 1982 for the treatment of hypertension [13,86,87].

**Figure 13:**
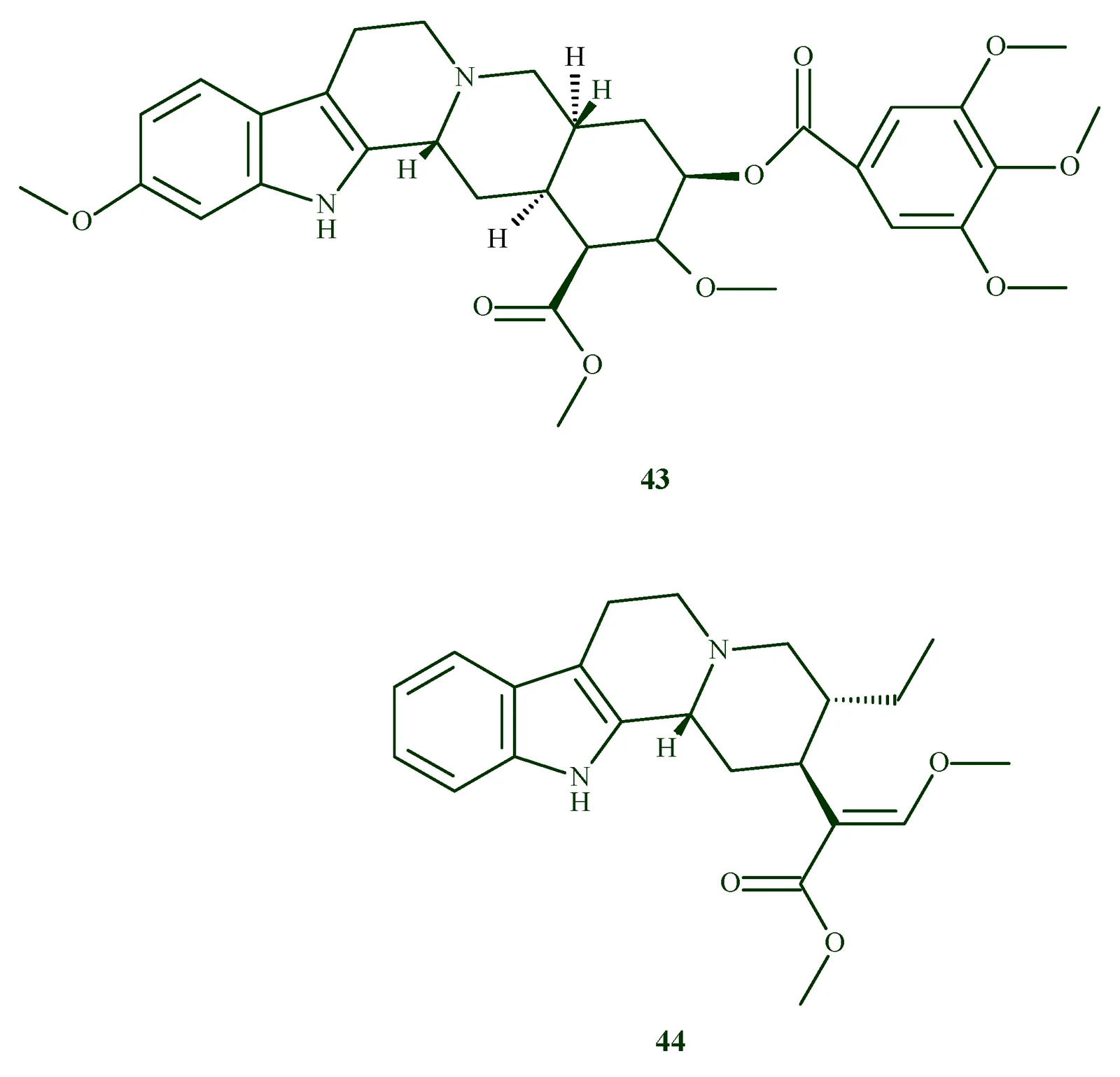
Structures **43** and **44**.

Reserpine (**43**) (Figure 13) is an indole alkaloid with antihypertensive and antipsychotic properties. Its pharmacological effects result from the depletion of catecholamines in peripheral sympathetic nerve endings [13,85]. Hirsutine (**44**) (Figure 13) is an indole alkaloid isolated from *Uncaria rhynchophylla* that has been reported to alleviate headache and dizziness associated with hypertension. It has also been reported to exhibit sedative and antiarrhythmic effects [13,88].

**Figure 14:**
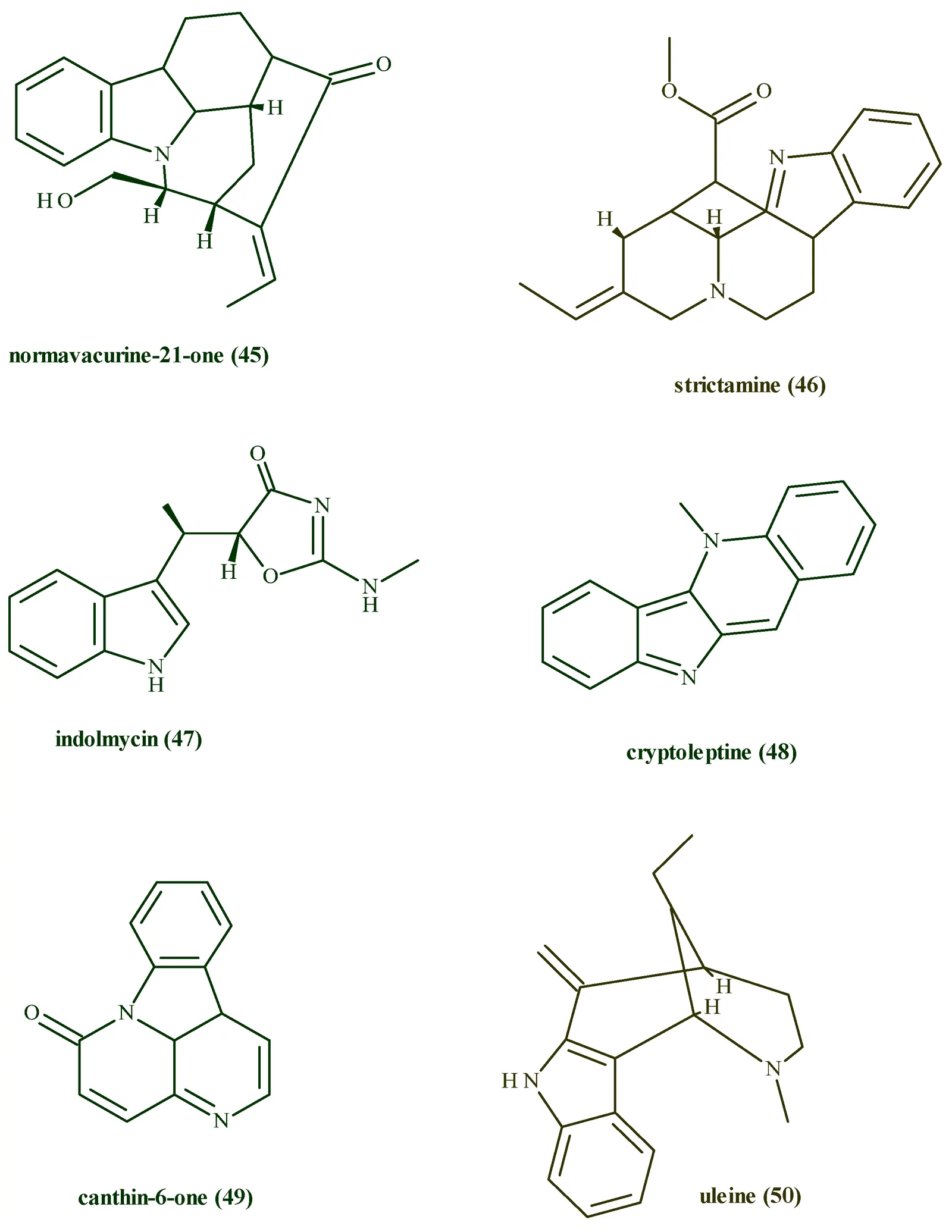
Structures **45**–**50**.

In addition to their therapeutic effects, including anticancer, antimicrobial, antidepressant, anti-inflammatory, anticholinergic, antimigraine, antiemetic, and antihypertensive activities, indole derivatives exhibit a broad range of antimicrobial properties. *Alstonia scholaris* is a plant used in traditional medicine for the treatment of malaria, gastrointestinal disorders, cancer, and jaundice. It contains numerous indole derivatives, particularly alkaloids, among which normavacurine-21-one (**45**) (Figure 14) and strictamine (**46**) (Figure 14) have been shown to inhibit the growth of *Enterococcus faecalis* [69]. 6-Bromoaplysinopsin (**23**) (Figure 14), isolated from *Smenospongia aurea*, exhibited antimalarial activity against *Plasmodium falciparum* [13,78]. Indolmycin (**47**) (Figure 14) has demonstrated antibacterial activity against *Staphylococcus aureus* and *Helicobacter pylori* [13,89,90]. Cryptolepine (**48**) (Figure 14) is an indoloquinoline derivative isolated from *Cryptolepis sanguinolenta* that has demonstrated antiplasmodial activity against chloroquine-resistant *Plasmodium falciparum* [13,44,91]. Canthin-6-one (**49**) (Figure 14), isolated from *Allium neapolitanum*, exhibited antibacterial activity against *Staphylococcus aureus* and *Mycobacterium* species [13,92]. Uleine (**50**) (Figure 14) is the major alkaloid isolated from the bark of *Aspidosperma parvifolium* and exhibits high in vitro antimalarial activity against a chloroquine-resistant strain of *Plasmodium falciparum* [13,93].

**Figure 15:**
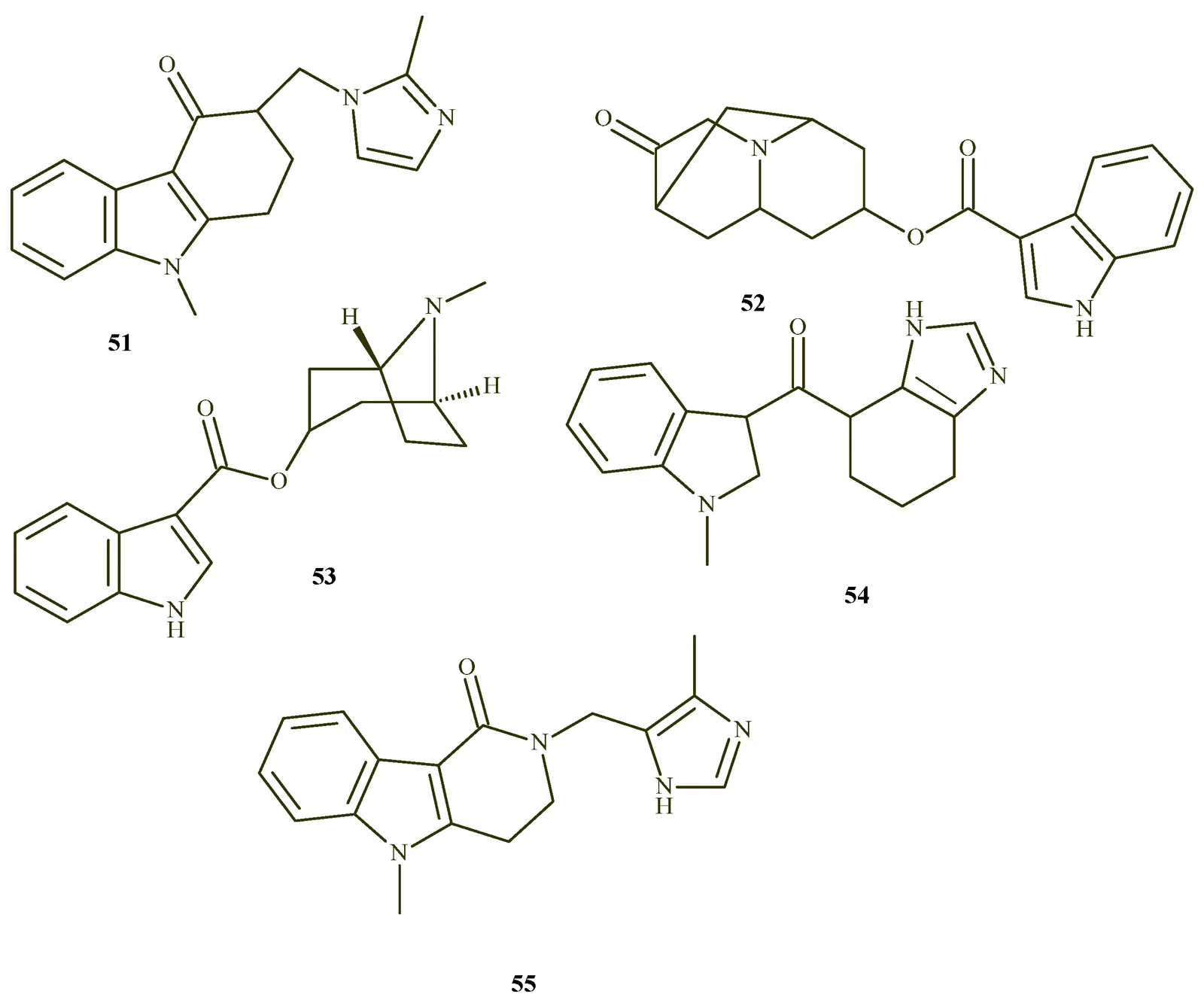
Structures **51**–**55**.

Effective pharmacological agents used for the treatment and prevention of nausea and vomiting include antiemetic drugs, such as ondansetron (**51**), dolasetron (**52**), tropisetron (**53**), ramosetron (**54**), and alosetron (**55**) (Figure 15). Ondansetron (**51**) is a 5-HT3 receptor antagonist commonly used to prevent nausea and vomiting associated with cancer chemotherapy, radiotherapy, and surgery. It was approved by the U.S. Food and Drug Administration (FDA) in 1991 [13,94]. The most common adverse effects associated with ondansetron include headache, allergies, and diarrhea [13,95]. Dolasetron (**52**) is used to prevent nausea and vomiting associated with chemotherapy and surgery [13,96]. It is a prodrug that is metabolized to hydrodolasetron, its pharmacologically active metabolite, primarily by carbonyl reductase. Tropisetron (**53**) is a 5-HT3 receptor antagonist used as an antiemetic and has also been investigated for analgesic effects in patients with fibromyalgia. Common adverse effects associated with tropisetron include headache, constipation, and dizziness [13,97]. Alosetron (**55**) is a selective 5-HT3 receptor antagonist that was approved by the FDA in 2000 for the treatment of women with diarrhea-predominant irritable bowel syndrome [13,98].

## 3. Therapeutic Goals of Indoles in Cancer Transformation

**Figure 16:**
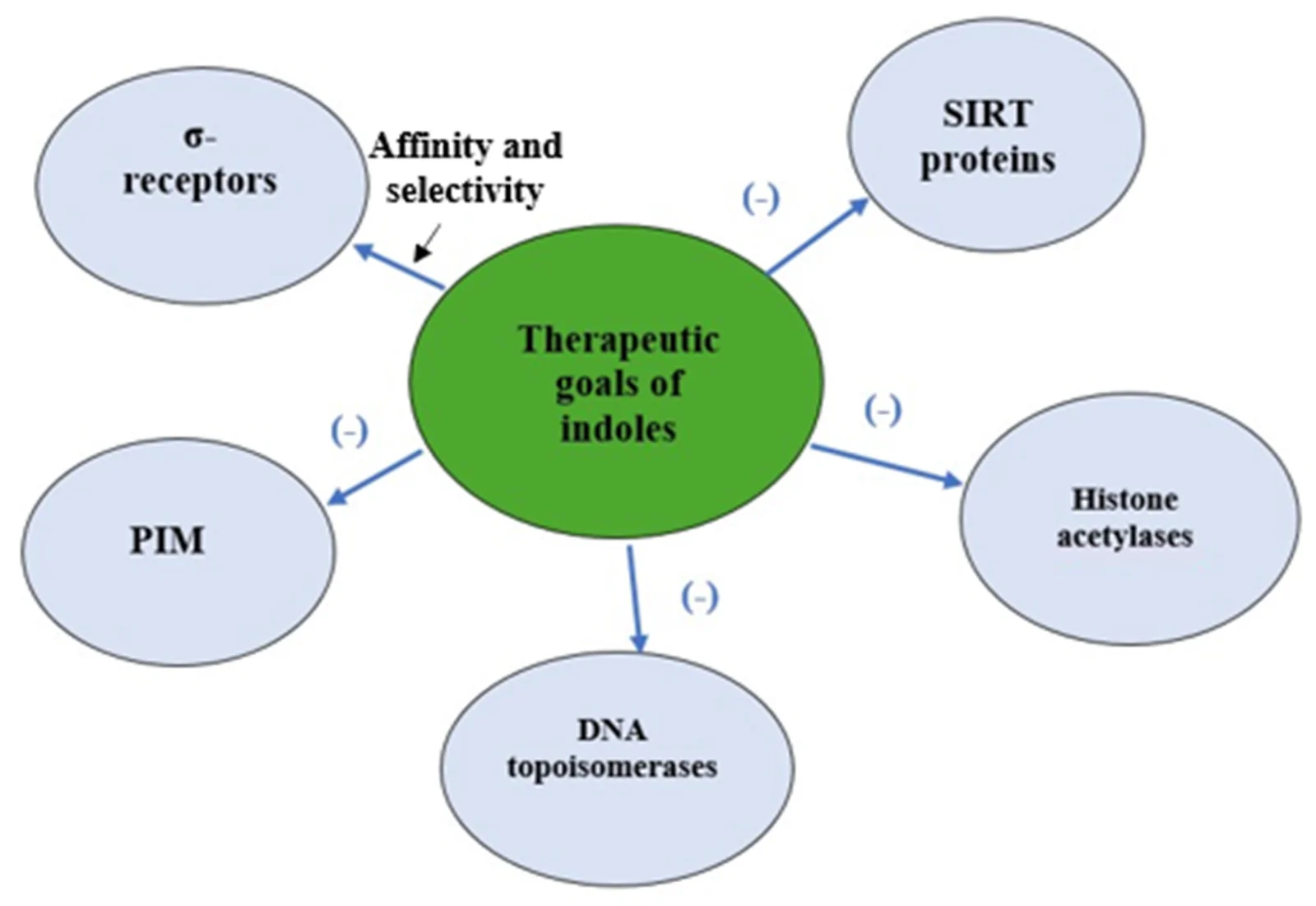
Molecular goals of indoles in cancer transformation (-) indicates inhibitory ability of indoles towards target molecules such as: SIRT (silent information regulator 2 proteins); histone acetylases; DNA topoisomerases; PIM (a serine/threonine kinase). Sigma (σ) receptors: affinity and selectivity of indoles).

Biological evaluation and studies investigating the anticancer mechanisms of indoles have revealed that these compounds exert their effects through various molecular pathways in cancer cells. Numerous indole derivatives have been described as inhibitors of tubulin polymerization, with the potential to interact with the colchicine-binding site [99,100]. Such compounds are referred to as colchicine-binding site inhibitors (CBSIs) because they interfere with microtubule formation and consequently induce cell-cycle arrest [101]. Other important therapeutic targets of anticancer indoles include histone acetyltransferases and deacetylases, sirtuins (SIRT proteins; silent information regulator 2 proteins), PIM kinases, DNA topoisomerases, and sigma (σ) receptors (Figure 16). For example, dysregulation of the balance between histone acetylation and deacetylation may contribute to carcinogenesis, as alterations in the activity of these enzymes can affect gene expression, DNA repair, and cell survival. SIRT proteins exhibit several enzymatic activities, including deacetylase and ADP-ribosyltransferase (ART) activities, and are dysregulated in various types of malignancies [102]. PIM kinases are involved in signaling pathways that regulate tumor cell proliferation and are frequently overexpressed in different types of cancer. Inhibition of DNA topoisomerases in tumor cells can interfere with DNA replication and transcription, thereby contributing to cell cycle arrest and cell death. Sigma-1 and sigma-2 receptors are considered potential targets for multitarget-directed ligands (MTDLs), allowing the simultaneous modulation of multiple molecular targets and potentially producing synergistic anticancer effects [13,103].

### 3.1. Histone Acetyltransferases and Deacetylases

Histone acetyltransferases (HATs) and histone deacetylases (HDACs) are two families of key enzymes that regulate gene expression and the cell cycle [2,13,104]. Acetylation of lysine residues in histone tails leads to chromatin relaxation and promotes gene transcription. Conversely, removal of acetyl groups from histone tails results in increased chromatin condensation and promotes gene silencing [2,13,105,106,107]. The balance between histone acetylation and deacetylation is essential for various cellular processes, including cell proliferation, cell cycle regulation, and apoptosis [2,13,108]. The human genome encodes 18 histone deacetylases, which are classified into four classes based on their structural characteristics, functions, subcellular localization, and expression patterns [2,109,110]. Increased HDAC expression has been observed in several types of cancer, including myeloid neoplasms and solid tumors. Therefore, HDACs represent promising molecular targets for anticancer therapy [2,104].

### 3.2. Kinases

**Figure 17:**
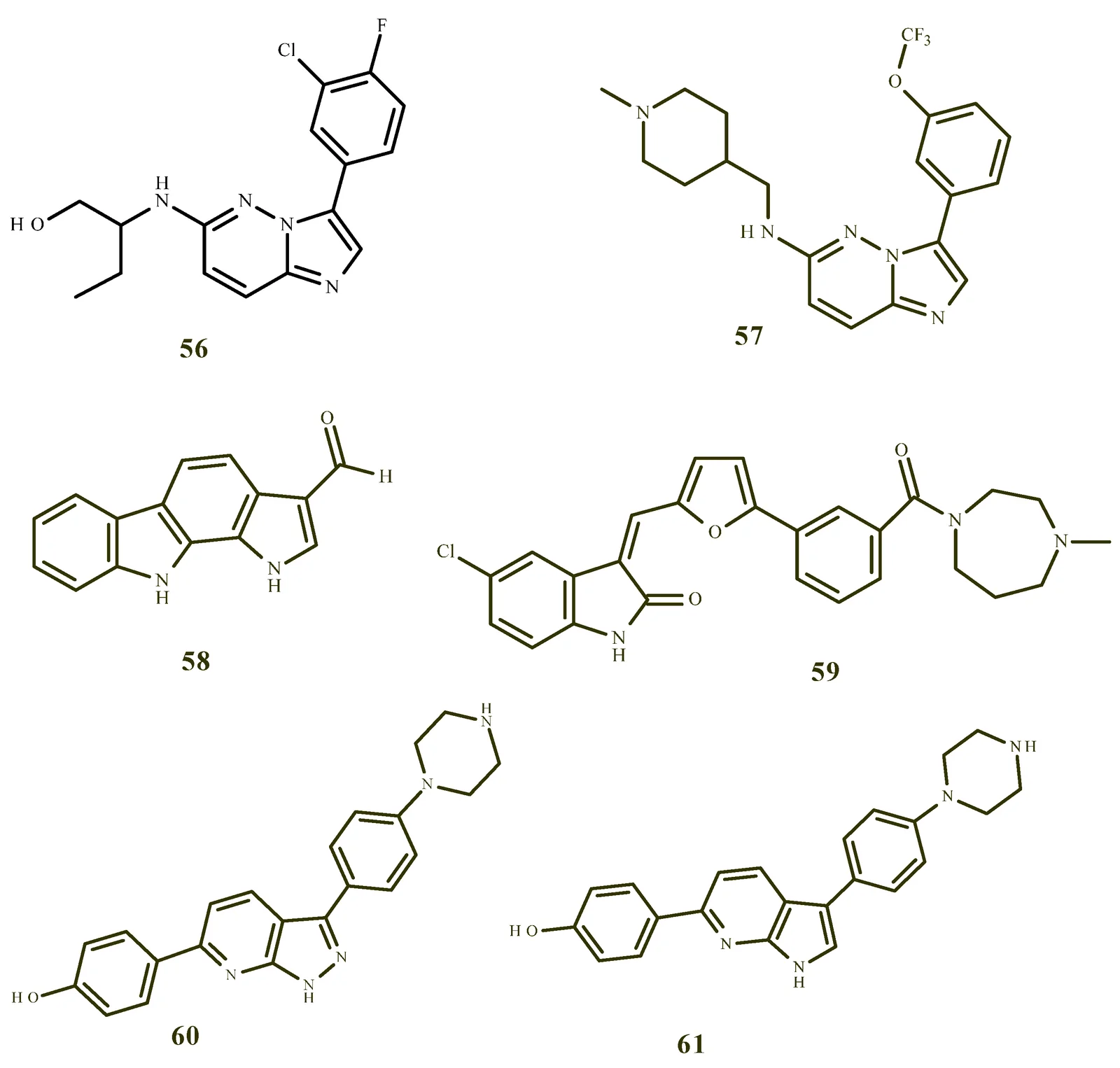
Structures **56**–**61**.

This subsection briefly describes selected kinases that are important molecular targets in anticancer therapy. PIM kinases are serine/threonine kinases that occur as three isoforms, PIM1, PIM2, and PIM3, which constitute a family of short-lived, homologous serine/threonine kinases [2,111,112,113]. PIM kinases respond to cytokines and influence the JAK/STAT signaling pathway. They play key roles in regulating cell survival, proliferation, differentiation, and apoptosis. PIM kinases respond to cytokines and influence the Jak/Stat pathway. They play a key role in controlling cell survival, proliferation, differentiation and apoptosis [2,114,115,116]. In various human cancers, including hematological malignancies and adenocarcinomas, dysregulated expression of PIM kinases has been identified, making these enzymes potential targets for anticancer drug development [2,117,118]. Several PIM kinase inhibitors have been investigated, including compounds **56** [2,119,120], **57** [2,121], **58** [2,122,123], **59** [2,124] (Figure 17). Compounds **58** and **59** are 3- and 5-substituted derivatives of compound **60** and exhibited anti-FLT3, anti-c-KIT, anti-PDGFR, and anti-KDR activities. Structure–activity relationship (SAR) studies demonstrated that compound **61** (Figure 17) is active against PIM kinases (serine/threonine kinases) while showing no significant activity against other kinases. The 2-pyrazine and 3-pyridine analogs at the C-5 position exhibited improved activity. Replacement of the phenylpiperazine moiety with 2-fluoroaminopyridine resulted in increased potency [2].

**Figure 18:**
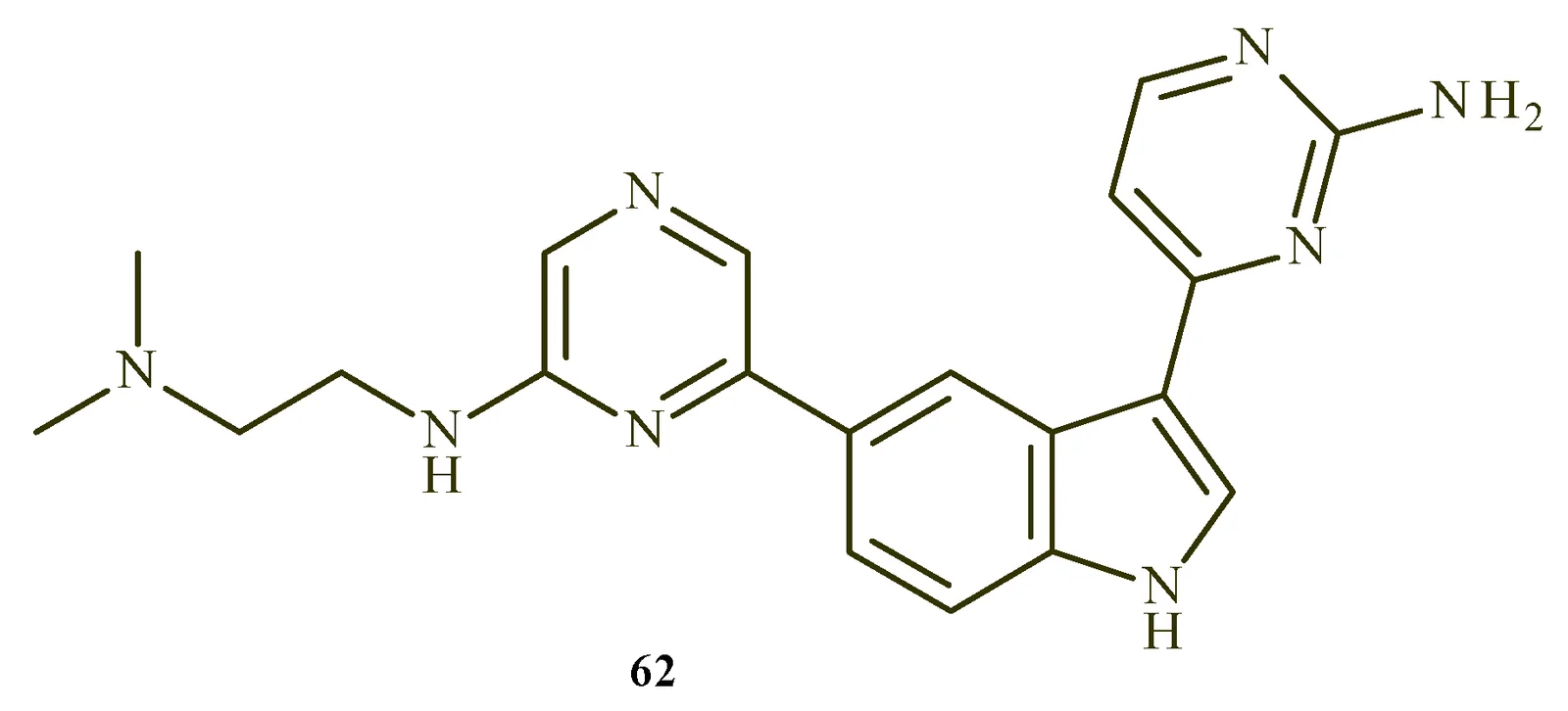
Structure **62**.

Compound **62** (Figure 18), which contains a dimethylaminoethylamine group, showed higher affinity for PIM1 and PIM3 kinases. Moreover, this compound exhibited good selectivity across a panel of 13 protein kinases, including serine/threonine kinases and receptor and non-receptor tyrosine kinases, with the exception of CDK2/cyclin A. Molecular modeling of the interaction between compound **62** and the ATP-binding site of PIM1 kinase suggests the formation of hydrogen bonds involving Pro123, Lys67, Glu121, Glu171, and Asn172 [2,125].

### 3.3. Other Proteins

In this subsection, we characterize the roles of sirtuins, sigma receptors, and DNA topoisomerases as potential targets in anticancer therapy.

**Figure 19:**
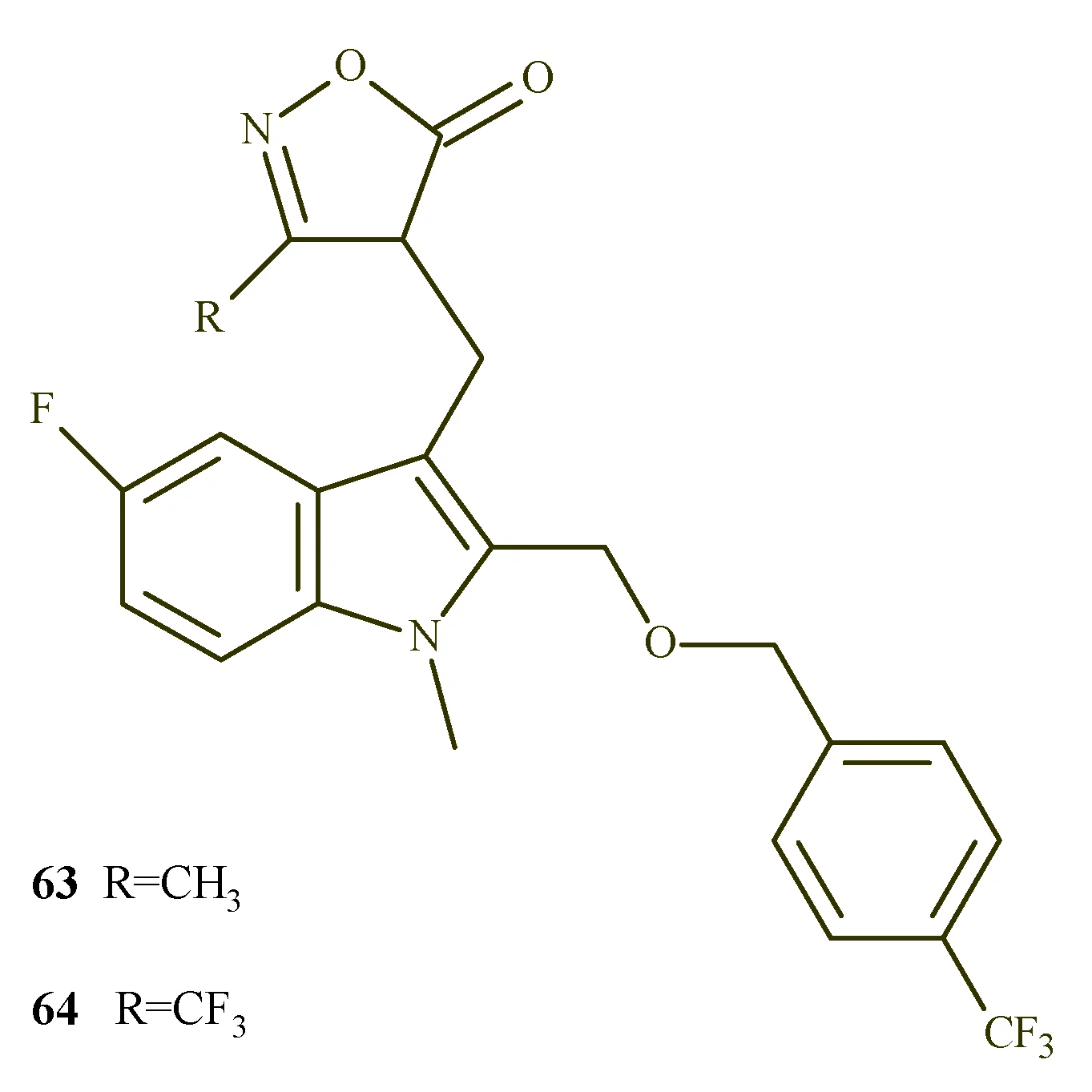
Structures **63** and **64**.

Sirtuins are a family of seven proteins (SIRT1–SIRT7) classified as class III histone deacetylases. Compounds **63** and **64** (Figure 19) exhibited high cytotoxicity against cancer cells of the MDA-MB-231, HT-29, and MCF-7 cell lines. Evaluation of the activity of compounds **63** and **64** against SIRT1 demonstrated promising inhibitory effects. Molecular docking of compound **63** at the catalytic site of SIRT1 revealed interactions with Phe297, Phe271, Asp348, and Ile347, as well as with a glutamine residue [2,126]. Elevated SIRT1 expression or activity has been reported in several types of cancer, including leukemia, prostate cancer, lung cancer, colorectal cancer, lymphoma, and soft tissue sarcomas, making SIRT1 a potential target for anticancer therapy [2,127,128,129]. It should also be noted that SIRT1 is involved in growth hormone signaling, which may contribute to the regulation of growth-related processes in humans [130].

**Figure 20:**
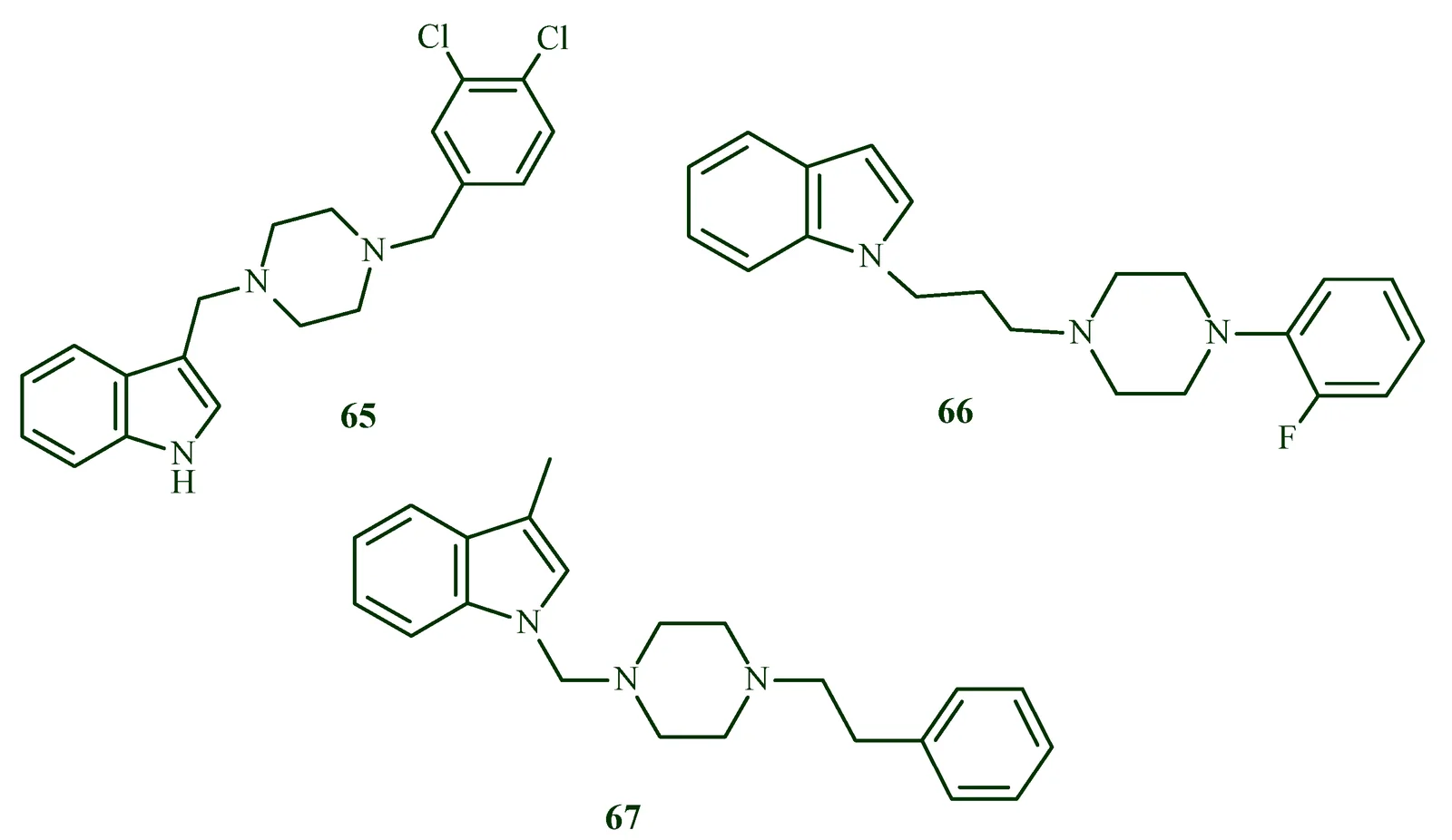
Structures **65**–**67**.

Sigma receptors, which are expressed in the central nervous system, are classified into two main subtypes, σ1 and σ2 receptors. Sigma receptors are involved in various biological and pathological processes, including depression [2,131,132], inflammation [2,133], anxiety [2,134], Alzheimer’s disease [2,135], cancer and epilepsy [2,135,136]. Studies assessing the affinity of selected compounds for sigma receptors have been conducted. Compound **65** (Figure 20) exhibited moderate affinity, whereas compound **66** (Figure 20), containing a phenylethyl substituent, showed high affinity for both σ1 and σ2 receptors. Compound **67** (Figure 20), bearing a phenylpiperazin-1-ylpropyl substituent, demonstrated high selectivity and affinity for the σ2 receptor [2].

**Figure 21:**
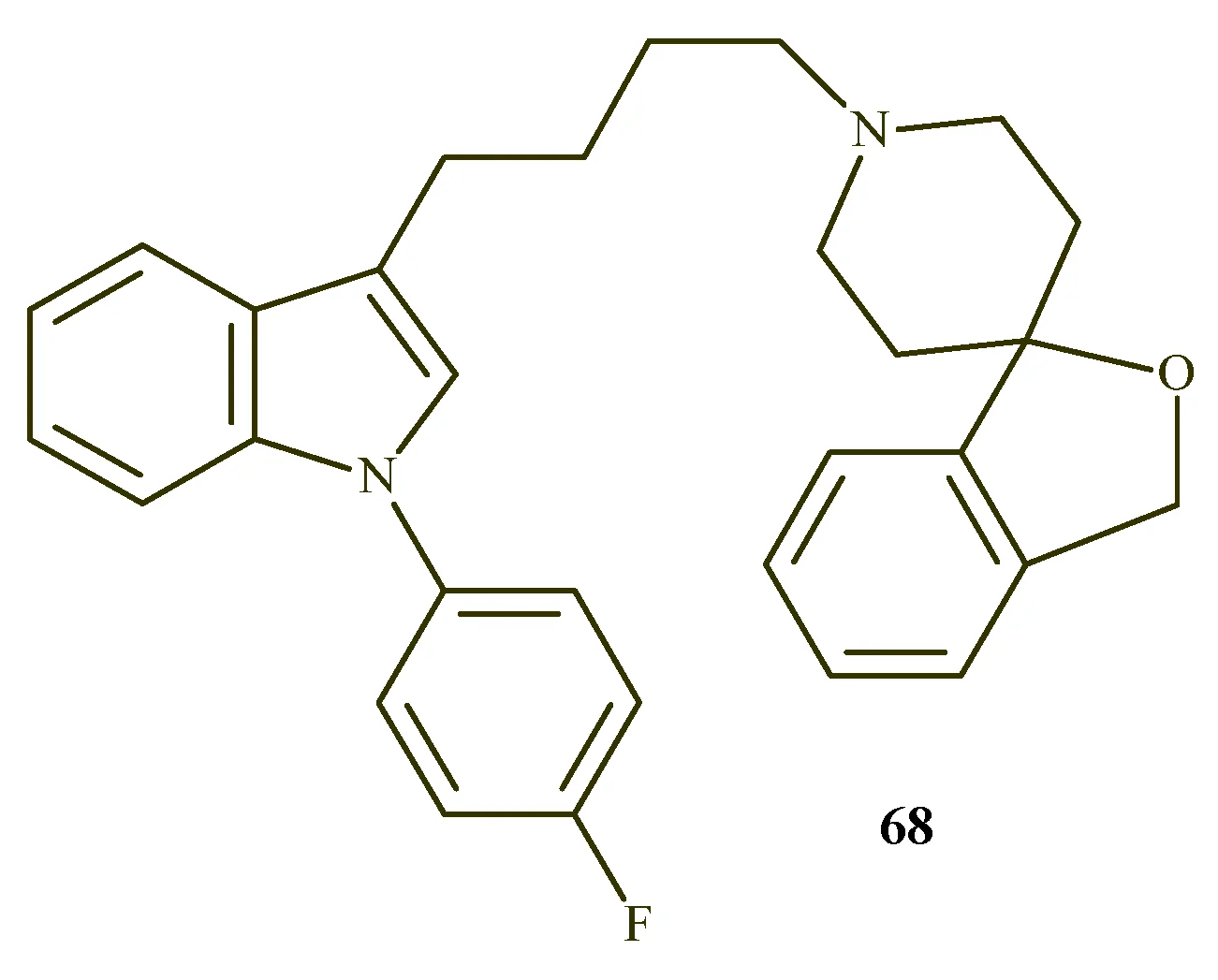
Structure of siramesine (**68**).

Siramesine (**68**) (Figure 21), a σ2 receptor agonist, has been investigated as a potential therapeutic agent for the treatment of depression and anxiety [2,137]. Its selectivity for the σ2 receptor has been attributed to the presence of an indole ring and a butylene linker connecting the indole moiety to the spirocyclic piperidine ring [2,138].

**Figure 22:**
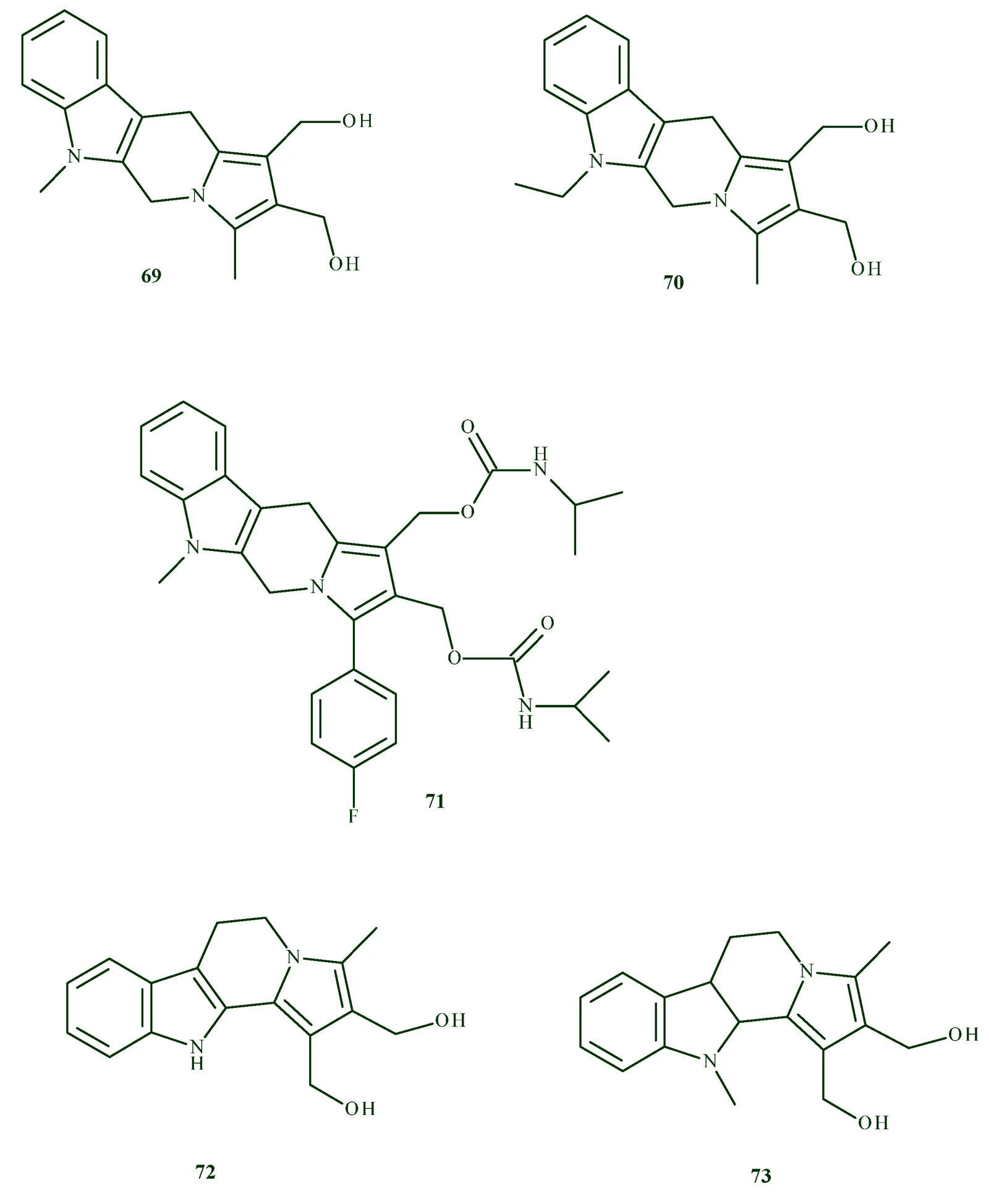
Structures **69**–**73**.

DNA topoisomerases introduce transient breaks in DNA strands to regulate DNA topology [2,139]. Cytotoxicity was evaluated against seven cell lines: HepG2 (human hepatocellular carcinoma), MCF-7 (human breast cancer), A375 (human malignant melanoma), HT1080 (human fibrosarcoma), LS174T (human colon cancer), BGC-823 (human gastric cancer), and A549 (human lung cancer), using the MTT assay. In particular, compound **69** (Figure 22) demonstrated good activity in human colon and lung cancer xenograft models. Moreover, evaluation of the therapeutic efficacy of compounds **70** and **71** (Figure 22) in the MX-1 mouse model of human breast cancer resulted in tumor inhibition of more than 99%. Cytotoxicity was evaluated against seven cell lines: HepG2 (human hepatocellular carcinoma), MCF-7 (human breast cancer), A375 (human malignant melanoma), HT1080 (human fibrosarcoma), LS174T (human colon cancer), BGC-823 (human gastric cancer), and A549 (human lung cancer), using the MTT assay. In particular, compound **69** (Figure 22) demonstrated good activity in human colon and lung cancer xenograft models. Moreover, evaluation of the therapeutic efficacy of compounds **70** and **71** (Figure 22) in the MX-1 mouse model of human breast cancer resulted in tumor inhibition of more than 99%.

Flow cytometric analysis of the effects of compounds **69**–**71** (Figure 22) on the cell cycle in CL141T human non-small cell lung cancer cells revealed an initial delay in S-phase progression, preceded by cell cycle arrest in the G2 phase. Experiments using supercoiled plasmid DNA followed by a relaxation assay with ATP-dependent topoisomerase II demonstrated that compound **69** inhibited both topoisomerase I and topoisomerase II, although its activity was lower than that of the respective positive controls: irinotecan for topoisomerase I and etoposide for topoisomerase II [2,140]. Compounds **72** and **73** (Figure 22) exhibited the highest cytotoxic activity. Evaluation of their effects on H460 lung cancer cells demonstrated induction of cell cycle arrest at the G2/M phase [2,141].

## 4. Thyroid Tumors

Thyroid cancer is the most common endocrine malignancy. Thyroid tumors occur more frequently in women and are often asymptomatic, with the highest incidence observed between 35 and 65 years of age. According to the International Agency for Research on Cancer (IARC), more than 237,000 new cases of thyroid cancer and approximately 2100 deaths attributable to the disease were reported worldwide in 2022. Thyroid cancer was among the three most frequently diagnosed cancers in individuals aged 15–39 years in 100 countries for women and 26 countries for men. Despite its relatively high incidence, thyroid cancer is associated with a low mortality rate [142].

The main types of TC include papillary, follicular, medullary, and anaplastic thyroid carcinoma. Papillary and follicular thyroid cancers, which are the most common types, are classified as DTCs and generally have the most favorable prognosis and the highest rates of curability. The initial treatment of thyroid cancer is primarily surgical and generally involves complete tumor resection, although the extent of surgery may vary depending on the tumor type and clinical characteristics, particularly in patients with papillary thyroid cancer (PTC) [143]. PTC accounts for approximately 80% of all DTCs [144,145]. Thyroid cancers are characterized by variable degrees of cellular proliferation, which may result from the accumulation of genetic alterations [144,146]. PTC is commonly associated with activating alterations in the *BRAF* and *RAS* genes and *RET*/*PTC* rearrangements, which can promote abnormal proliferation and survival of thyroid follicular cells.

Follicular thyroid cancer (FTC) is the second most common type of thyroid cancer after PTC. FTC accounts for approximately one-third of all DTCs [147,148]. The major oncogenic alterations in FTC include activating mutations in the *RAS* family of genes (*NRAS*, *HRAS*, and *KRAS*) and *PAX8*–*PPARγ* rearrangements. *RAS* mutations are detected in a substantial proportion of FTC cases. In addition, molecular alterations, including mutations and copy-number changes affecting genes involved in the PI3K/AKT pathway, have been identified in FTC. Alterations in *PIK3CA* are relatively common in FTC. In some cases, *PTEN* function is also impaired through inactivating mutations or epigenetic mechanisms [147,148,149,150]. The treatment of DTCs generally includes surgical resection, followed, when indicated, by radioactive iodine (RAI) therapy and thyroid hormone replacement with levothyroxine.

Poorly differentiated thyroid cancer (PDTC), including oncocytic cancer, is characterized by the absence of the characteristic nuclear features of differentiated thyroid cancer, increased mitotic activity, and tumor necrosis. Patients with PDTC often present with vascular invasion, lymph node metastases, extrathyroidal extension, and, in some cases, distant metastases [147,151,152]. *RAS* and *BRAF* mutations have been reported in approximately one-third of PDTC cases [147,153]. Mutations in the *TERT* promoter and *TP53* are characteristic molecular alterations in PDTC and are associated with tumor aggressiveness; these mutations may co-occur with *RAS* and *BRAF* mutations [154,155]. Other genetic alterations, including *CTNNB1* and *EIF1AX* mutations and *ALK* rearrangements, have also been reported in PDTC [154,156]. Somatic copy-number alterations are common in PDTC and include gains of chromosome 1q and losses of chromosomes 1p, 13q, 15q, and 22q [147,154]. Anaplastic thyroid cancer (ATC) is an undifferentiated thyroid cancer characterized by extremely rapid and aggressive growth. The disease progresses rapidly and is associated with a poor prognosis and a high mortality rate. ATC predominantly affects older individuals, with the majority of cases occurring in patients over 50 years of age [157].

## 5. Description of the Wnt Signaling Pathway and Its Involvement in Thyroid Physiology/Pathology

**Figure 23:**
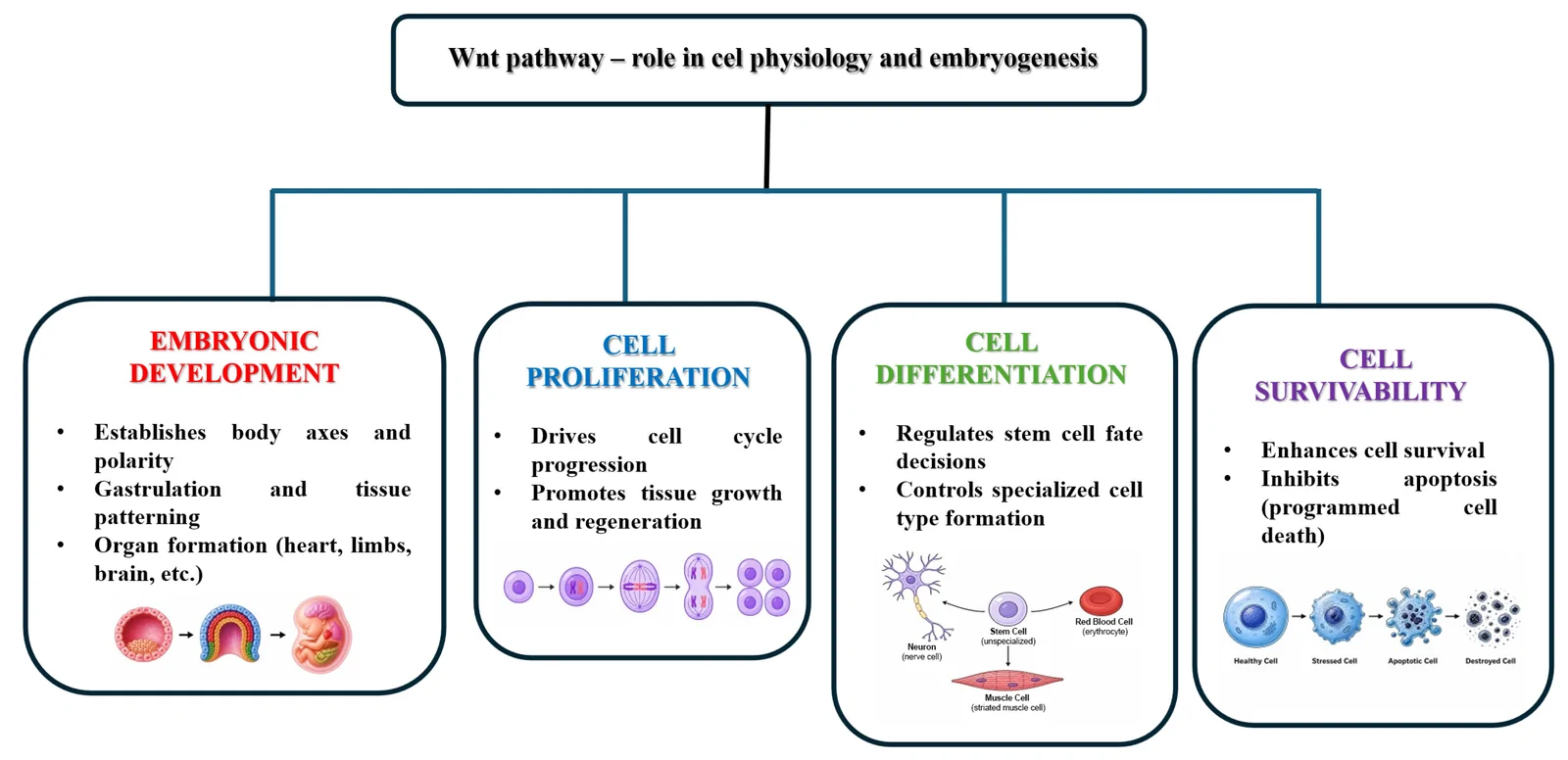
Role of the Wnt signaling pathway in cell physiology.

The Wnt pathway is an important signaling pathway in cell biology and is essential for proper morphogenesis, cellular differentiation, survival, and proliferation of cells (Figure 23). The pathway was first described in the 1970s and 1980s [158]. Dysregulation of this pathway is associated with the pathogenesis of numerous diseases, including cancers, neurodegenerative disorders and bone diseases [159,160]. Wnt/β-catenin signaling plays a crucial role in carcinogenesis by regulating the expression of genes involved in proliferation, cell survival, invasion, and metastasis [161]. Mutations in genes encoding components of the Wnt pathway, such as APC (Adenomatous Polyposis Coli) or β-catenin (CTNNB1), lead to the stabilization of β-catenin in the cytoplasm and its translocation to the nucleus, where it activates the transcription of target genes [161]. Aberrant activation of this pathway has been observed in many types of cancers, including colorectal cancer, hepatocellular carcinoma (HCC), and pediatric tumors [161,162,163]. It should be emphasized that the Wnt pathway is essential for the proper functioning of the nervous system, regulating neurogenesis, synaptogenesis, and synaptic plasticity [164]. Dysfunction of this pathway is involved in the pathogenesis of neurodegenerative diseases such as Parkinson’s disease (PD), Alzheimer’s disease (AD) and Huntington’s disease (HD) [164,165]. Potential mechanisms underlying this dysregulation include accumulation of toxic proteins, mitochondrial dysfunction, and increased oxidative stress, contributing to neuronal degeneration. For example, in AD, the Wnt/β-catenin pathway is often inhibited, which exacerbates amyloid and tau pathology [164]. Other important roles of this pathway in human physiology include participation in bone metabolism, regulation of osteogenesis, bone homeostasis, and osteoblast differentiation [166]. Mutations in the LRP5 gene (low-density lipoprotein receptor-related protein 5) (Wnt-related gene), can lead to increased or decreased bone mass [166]. Dysregulation of the Wnt pathway is associated with bone diseases, such as osteoporosis, sclerosteosis, van Buchem disease [167,168].

**Figure 24:**
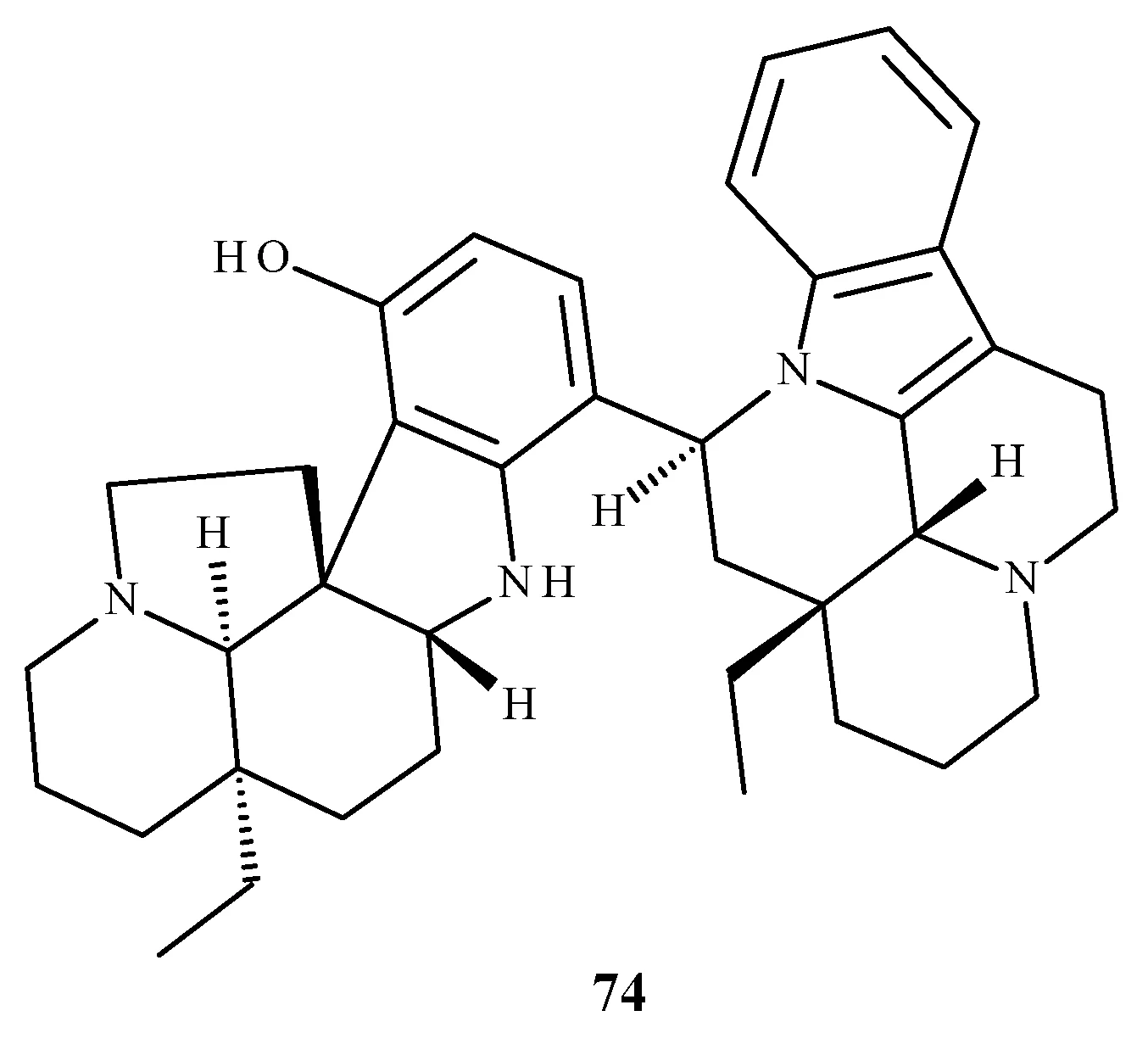
Bisleuconothine A (**74**).

Generally, the Wnt pathway is divided into two types: canonical (β-catenin-dependent pathway) and non-canonical (β-catenin-independent pathway). Additionally, cytogenetic studies confirmed the presence of two non-canonical Wnt pathways, which are independent of β-catenin and involved in the regulation of cell movement. The non-canonical Wnt pathways are further divided into two main branches: the Wnt/Ca^2+^ pathway and the Wnt/planar cell polarity (PCP) pathway. Both are independent of β-catenin and involved in the regulation of cell movement and polarity. The Wnt/Ca^2+^ pathway regulates intracellular calcium signaling, inflammation, and apoptosis, whereas the Wnt/PCP pathway modulates cell migration and plays a key role in the regulation of cell polarity. In contrast, the canonical Wnt pathway depends on β-catenin and regulates the transcriptional activity of T-cell factor/lymphoid enhancer factor (TCF/LEF). Non-canonical Wnt pathways are independent of β-catenin and TCF/LEF and are thought to play important roles in cytoskeletal rearrangement and neuronal migration [169,170]. Numerous studies have supported the hypothesis that Wnt family proteins play a crucial role in cancer transformation, primarily by promoting the self-renewal capacity of cells and inhibiting their differentiation. It has been demonstrated that cancer cells with increased Wnt-1 expression are resistant to apoptosis, as Wnt-1 signaling inhibits the release of cytochrome c into the cytosol and suppresses the proteolytic activity of caspase-9 [171]. Therefore, the use of Wnt pathway inhibitors as targeted therapeutic agents may significantly reduce cancer cell proliferation, growth, and migration. The use of Wnt pathway inhibitors as activators of targeted therapy could significantly reduce the growth of cancer cells, their division and even migration. The canonical Wnt pathway requires β-catenin and mediates the activation of TCF/LEF-dependent transcription [171]. B-Catenin is also a component of adherens junctions, where, together with E-cadherin, it contributes to the stabilization and organization of the cytoskeleton. The JNK (c-Jun N-terminal kinase) pathway is also involved in non-canonical Wnt signaling. The Jnk (c-Jun amino-terminal kinase) pathway is also involved in the non-canonical type of Wnt pathway [172]. It is worth mentioning that the bisindole alkaloid bisleuconothine A (**74**) (Figure 24), isolated from the bark of *Leuconotis griffithii*, has been shown to inhibit Wnt signaling in human colorectal cancer cell lines (HCT116 and SW480) both in vitro and in vivo [173].

The thyroid gland is essential for maintaining systemic homeostasis through the production and secretion of thyroid hormones. The differentiated epithelial phenotype of the thyroid gland is characterized by structural and functional polarization of the cell surface into apical and basolateral domains, as well as by the formation of intercellular junctions that mediate cell–cell adhesion. Cell polarity plays a key role in maintaining the structural organization of thyroid follicles, and alterations in cell polarity may occur during cellular transformation. The presence of tight junctions between thyroid epithelial cells is important for maintaining epithelial integrity and the structural organization of thyroid follicles. Claudins and occludin play important roles in maintaining epithelial cell integrity and phenotype [174]. These proteins are integral components of tight junctions, although they belong to distinct protein families. Claudins are the major structural components of tight junctions and are essential for the formation and regulation of the paracellular barrier, whereas occludin contributes to the organization and regulation of tight junction structure and function. Under pathological conditions, including inflammation, cancer, and infection, tight junction integrity may be disrupted [175]. Thyrotropin (TSH) secretion is primarily regulated by the hypothalamic–pituitary–thyroid axis through a negative feedback mechanism involving circulating thyroid hormones. The role of Wnt signaling in thyroid carcinogenesis is supported by evidence from familial cancer syndromes, particularly familial adenomatous polyposis (FAP), which is associated with an increased risk of TC. FAP is caused by germline mutations in the *APC* gene, a key negative regulator of the canonical Wnt/β-catenin signaling pathway. Patients with FAP, as well as those with Gardner syndrome and Turcot syndrome, may have an increased risk of TC, particularly papillary thyroid carcinoma (PTC), suggesting a possible association between *APC* mutations, aberrant Wnt signaling, and thyroid tumorigenesis [176,177].

However, the genetic alterations characteristic of TC are complex, and dysregulation of the Wnt pathway has been reported across different TC subtypes. It has been shown that the metastatic potential of papillary thyroid carcinoma (PTC) is associated with decreased E-cadherin expression and increased β-catenin levels [178,179]. There are three main genetic alterations in PTC: the *BRAFV600E* mutation, *RET*/*PTC* fusion, and RAS mutations. Recent studies have shown that all three genetic subtypes ultimately stimulate the Wnt signaling pathway through distinct tumor-promoting mechanisms, leading to dysregulation of the physiological functions of this pathway. The Wnt signaling pathway frequently interacts with other signaling pathways, such as MAPK, RET/PTC, and Akt/MAPK, which are involved in the initiation and progression of cancer. Crosstalk between the Wnt and MAPK/ERK pathways has been demonstrated mainly in melanoma and lung cancer. Additionally, transcriptomic analyses have revealed that *BRAFV600E* mutations can activate β-catenin, which may contribute to resistance to immunotherapy in PTC. Moreover, β-catenin can be stabilized through phosphorylation at sites other than those targeted by GSK3β, a process that may be mediated by RET/PTC rearrangements. Inhibition of GSK3β by Akt/MAPK signaling promotes the accumulation of β-catenin in the nucleus and activation of cyclin D1 (CCND1), thereby contributing to the proliferation of PTC cells. RET/PTC fusion stimulates Wnt signaling by promoting β-catenin stabilization [178,179,180,181,182]. High *WNT10A* expression in PTCs has been reported to enhance β-catenin signaling [178]. Data obtained from the database showed increased expression of WNT10A, CTNNB1, and CCND1, whereas AXIN2 expression was decreased, suggesting activation of Wnt signaling in PTC. As mentioned above, the canonical Wnt pathway is dependent on the levels of β-catenin. In the absence of Wnt signaling, free cytoplasmic β-catenin that is not bound to E-cadherin is targeted for degradation by the β-catenin destruction complex, which consists primarily of APC, Axin, CK1, and GSK3β. CK1 and GSK3β phosphorylate β-catenin, thereby promoting its recognition by β-TrCP (beta-transducin repeat-containing E3 ubiquitin protein ligase), an E3 ubiquitin ligase that mediates the ubiquitination of β-catenin and targets it for proteasomal degradation.

The Wnt signaling cascade is activated when Wnt ligands bind to the Frizzled (FZD) receptors and the LRP5/6 coreceptors, leading to the recruitment and activation of Dishevelled (DVL) and inhibition of the β-catenin destruction complex. Consequently, β-catenin accumulates in the cytoplasm, translocates to the nucleus, and forms a complex with TCF/LEF transcription factors, thereby promoting the expression of target genes such as *CCND1* and *MYC*. Dickkopf-1 (Dkk1) is a negative regulator of Wnt signaling that binds to LRP5/6 and inhibits the formation of the Wnt–FZD–LRP5/6 receptor complex.

The literature provides evidence for the role of genetic alterations leading to activation of the MAPK pathway in PTC. These include predominantly *BRAF* V600E mutations, *BRAF* rearrangements, *RET/PTC* rearrangements, and *RAS* oncogene mutations. Increasing evidence also indicates that oncogenic alterations can influence the PI3K pathway. A PAX8/PPARγ-induced transformation mechanism has been identified in a significant proportion of these tumors [180].

APC and β-catenin mutations, as well as RET/PTC rearrangements, occur in the cribriform-morular variant of TC [178,183,184,185]. These rearrangements lead to constitutive activation of the RET oncogene and stimulation of the MAPK and PI3K/AKT pathways [178,186,187]. Recent studies indicate that RET/PTC fusions also activate the Wnt pathway by phosphorylating β-catenin at the tyrosine residue Y654, thereby inhibiting its turnover [178,180,182]. It is worth mentioning that the molecular pathogenesis of anaplastic thyroid carcinoma (ATC), a highly aggressive form of thyroid carcinoma, is mainly associated with dysfunction of the p53 pathway and mutations affecting β-catenin [156]. Moreover, ATC is characterized by enhanced Wnt/β-catenin signaling, as demonstrated by analyses of thyroid resection specimens from patients. In addition, increasing evidence supports the involvement of other signaling pathways, including MAPK, in PTC [188].

Although PTC generally has an excellent prognosis, several challenges remain unresolved by clinicopathological studies. Differentiated thyroid cells form follicular structures surrounding the lumen, within which thyroid-specific transcription factors and other thyroid-specific proteins are expressed [174,189,190]. The proliferation and differentiation of thyroid cells are regulated by TSH, whose binding to its receptor leads to activation of the cAMP/PKA signaling cascade. TSH has also been shown to participate in the regulation of the PI3K/AKT pathway [191].

**Figure 25:**
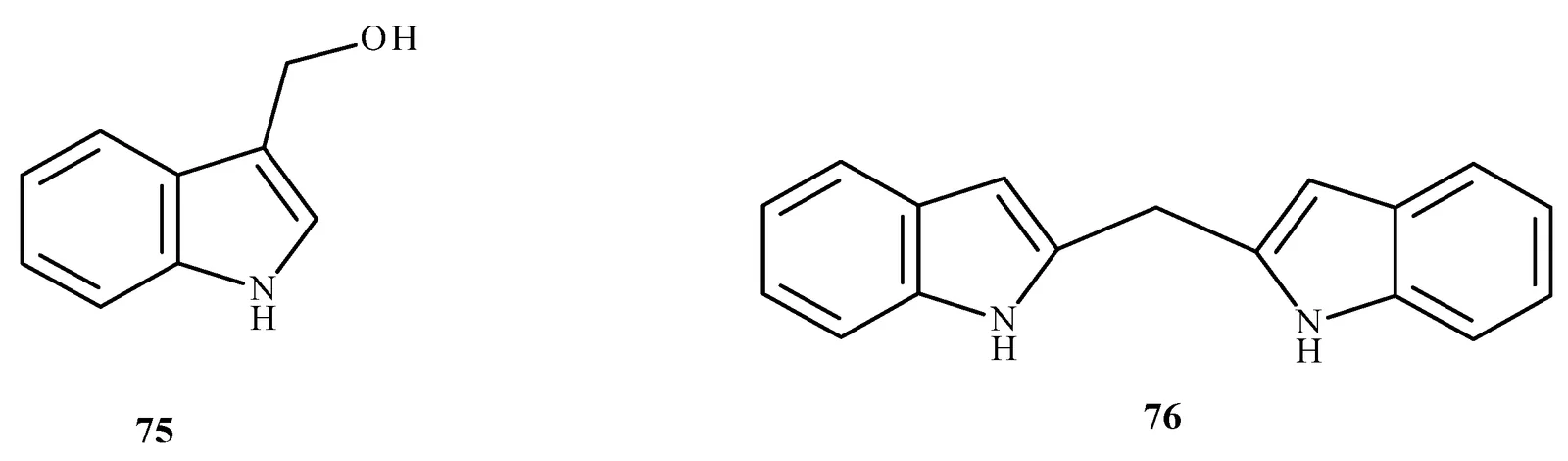
Structures **75** and **76**.

Vegetables from the *Brassicaceae* family, including cabbage, brussels sprouts, broccoli, and cauliflower, contain substantial amounts of glucosinolates, including glucobrassicin and neoglucobrassicin [192,193]. Glucobrassicin is hydrolyzed by the enzyme myrosinase, which is physically separated from glucosinolates in intact plant cells. When plant tissues are disrupted, for example, during chewing or food preparation, myrosinase is released and comes into contact with glucosinolates. Hydrolysis of glucobrassicin results in the formation of indole-3-carbinol (I3C) (**75**) (Figure 25) [194]. Compound **75** is unstable under the acidic conditions of the human stomach, where it undergoes condensation reactions leading to the formation of various oligomeric and polycyclic derivatives [195]. 3,3′-Diindolylmethane (DIM) (**76**) (Figure 25), a major product of I3C metabolism, has been reported to induce apoptosis in ML-1 and CGTH-W-1 cells, which are FTC cell lines. The level of apoptosis induced by DIM was 4–5-fold higher than that observed in untreated cells. Cell cycle arrest at the G1 phase was suggested to be one of the mechanisms contributing to DIM-induced cell death. In the 8505-C cell line, derived from human anaplastic thyroid cancer (ATC), neither **75** nor **76** produced a significant effect on apoptosis. The most pronounced effects of these compounds were observed in primary cultures of goiter-derived thyroid cells. These findings suggest that DIM may exert antiproliferative effects in FTC cells. Its potential advantages include relatively high stability, low toxicity, and favorable cellular uptake under experimental conditions [192]. At lower concentrations, compound **75** prevented Fenton reaction-induced lipid peroxidation in porcine ovary and kidney homogenates. In contrast, higher concentrations of **75** exhibited pro-oxidative effects [196]. Clinical trials investigating compound **75** have reported that its recommended dose for a 70 kg person should be from 2.9 to 5.7 mg/kg. Moreover, compound **75** has been approved by the FDA as a dietary supplement [197], but it has not been approved for use as a nutritional supplement in Europe. A safety assessment is required in accordance with the new regulations governing dietary supplements for approval by the European Commission [197,198].

## 6. Conclusions

Despite the limited number of studies investigating the effects of indole compounds on TC cells, this review provides an overview of indole compounds and their potential interactions with the Wnt signaling pathway. Indoles represent a diverse and widely studied class of naturally occurring compounds and their synthetically modified derivatives. Structural modification of these compounds has enabled the development of derivatives that have been used in the treatment of various diseases, including cancer. However, there is a lack of experimental studies directly evaluating the effects of indole compounds on the Wnt pathway in TC cells. The Wnt signaling pathway is involved in numerous developmental and physiological processes; however, its dysregulation may lead to pathological conditions, including cancer. This signaling pathway is altered in several types of cancer and is associated with tumor progression and metastasis.

Many of the indole compounds described in this review have been investigated in vitro. However, the results obtained from such studies cannot necessarily be extrapolated to in vivo conditions. Moreover, chemical compounds exhibiting therapeutic activity may also exert toxic effects at higher concentrations. The therapeutic application of naturally occurring compounds may be limited by their physicochemical properties, including poor solubility, which can reduce their bioavailability. Consequently, insufficient bioavailability may decrease the therapeutic efficacy of these compounds. Furthermore, interactions between different biochemical signaling pathways may significantly influence the response to a given therapy and contribute to the development of drug resistance.

In summary, this review characterizes indole derivatives in terms of their potential medicinal properties and provides examples of their applications in this field, with particular emphasis on their potential effects on the Wnt signaling pathway in TC. Special attention is given to PTC, as it is the most common histological type of thyroid cancer worldwide. Targeting the Wnt/β-catenin pathway with indole derivatives represents a promising therapeutic strategy. Given the critical role of this pathway in the development and progression of TC, its modulation by naturally occurring compounds such as indoles may provide opportunities for the development of novel therapeutic approaches. The ability of certain indole derivatives to inhibit cellular proliferation and invasion while promoting apoptosis further supports their potential as candidates for targeted therapy. Further research into the precise mechanisms of action of individual indole compounds, as well as their potential synergistic effects with existing anticancer treatments, may contribute to the development of more effective and less toxic therapeutic strategies for TC and other malignancies driven by aberrant Wnt signaling.

## Data Availability

No new data were created or analyzed in this study. Data sharing is not applicable.

## Abbreviations

The following abbreviations are used in this manuscript:

TC	Thyroid cancer
WNT10A	Wnt Family Member 10A
WHO	World Health Organization
EU	European Union
EMT	Epithelial–mesenchymal transition
DTCs	Differentiated thyroid cancers
PTCs	Papillary thyroid cancers
FTC	Follicular thyroid cancer
FDA	Food and Drug Administration
LPO	Lipid peroxidation
ASMT	Acetyl serotonin methyltransferase
AANAT	Aralkylamine N-acetyltransferase
MT1	Melatonin receptor
SGC-7901	Human gastric cancer cell line
CAPE	Caffeic acid phenethyl ester
HSV1, HSV2	Human herpesvirus
H_3_N_2_	Virus A/Hanfang/359/95
HIV-1	Human immunodeficiency virus 1
NNRT1	Non-nucleoside reverse transcriptase inhibitor
P-388	Murine (mouse) lymphoblastic leukemia model
P450	Cytochrome enzyme
CYP3A4	Cytochrome enzyme
5HT2A	Serotonin receptor 2A
5HT2C	Serotonin receptor 2C
MOA-A	Monoamine oxidase A
RIMAs	Reversible Monoamine Oxidase Inhibitors
5-HT1B/1D	Serotonin receptor 1B/1D
ACE	Angiotensin-converting enzyme
AT1	Angiotensin II type 1 receptor
5HT3	Serotonin receptor 3
SIRT	Sirtuin proteins
ART	ADP-ribosyltransferase
ADP	Adenosine diphosphate
PIM	Serine/threonine kinases
MTDL	Multi-target directed ligand
HATs	Histone acetyltransferases
HDACs	Histone deacetylases
PIM1, PIM2, PIM3	Serine/threonine kinases
Jak/Stat	Janus kinase (JAK)-signal transducer of activators of transcription (STAT) pathway
anti-Flt-3	FMS-like tyrosine kinase-3
anti-c-Kit	Tyrosine-protein kinase KIT
anti-PDGFR	Platelet-Derived Growth Factor Receptor
anti-Kdr	Protein kinase insert domain receptor
SAR	Structure–activity relationship
CDK2/cyclin A	Protein complex that activates cyclin-dependent kinase 2
Pro123	Proline in position 123
Lys67	Lysine in position 67
Glu121	Glutamic acid in position 121
Glu171	Glutamic acid in position 171
Asn172	Asparagine in position 172
MDA-MB-231	Human breast cancer cell line
HT-29	Human colon cancer cell line
MCF7	Human breast cancer cell line
SIRT1	Sirtuin 1
Phe271	Phenylalanine in position 271
Phe297	Phenylalanine in position 297
Asp348	Aspartic acid in position 348
HepG2	Human liver cancer cell line
A375	Human malignant melanoma cell line
HT1080	Human fibrosarcoma cell line
LS174T	Human colonic cancer cells
BGC-823	Human gastric cancer cell line
A549	Human lung cancer cell line
MTT	A colorimetric assay for assessing cell metabolic activity
IARC	International Agency for Research on Cancer
SOD	Superoxide dismutase
CAT	Catalase
MT1/MT2	Melatonin receptors
GSK3β	Glycogen synthase kinase-3 β
GR	Glucocorticoid receptor
GPx	Glutathione peroxidase
MX-1	Human breast carcinoma cell line
CL141T	Human non-small cell lung cancer cell line
ATP	Adenosine triphosphate
TopI	Topoisomerase I
TopII	Topoisomerase II
BRAF	Human gene that encodes a protein called B-Raf
RAS	Oncogene
RET/PTC	Rearrangement of the RET gene
RAI	Radioactive iodine
cPTC	Cystic papillary thyroid carcinoma
PAX8/PPARy	A fusion gene, encoding the PPFP fusion protein
PI3K/Akt	An intracellular signaling pathway important in regulating the cell cycle
PIK3CA	Phosphatidylinositol-4,5-bisphosphate 3-kinase catalytic subunit alpha
PTEN	Phosphatase and tensin homolog deleted on chromosome ten
PDTC	Poorly differentiated thyroid carcinoma
CTNNB1	Catenin β 1
Wnt Wingless and Int-1	Wnt signaling pathway
PD	Parkinson’s disease
AD	Alzheimer’s disease
HD	Huntington’s disease
Wnt/β-catenin	Canonical Wnt pathway; β-catenin-dependent pathway
Non-canonical Wnt pathway	β-catenin-independent pathway
Wnt/Ca^2+^ pathway	Non-canonical Wnt pathway
Wnt/PCP pathway	Wnt/planar cell polarity pathway (non-canonical Wnt pathway)
TERT	Telomerase reverse transcriptase
TP53	Tumor protein p53
EIF1AX	Eukaryotic translation initiation factor
ALK	Anaplastic lymphoma kinase
ATC	Anaplastic thyroid carcinoma
TCF	T cell factor
Wnt-1	Wnt family member 1
Tcf/Lef	T cell factor/lymphoid enhancer factor family
Jnk	c-Jun N-terminal kinase
HCT116	Human cancer colorectal cell line
SW480	Human cancer colorectal cell line
JAM	Junctional adhesion molecule
TSH	Thyroid stimulating hormone
FAP	Familial adenomatous polyposis
BRAFV600E	mutation of gene BRAF
APC	Adenomatous polyposis coli
CCND1	Cyclin D1
AXIN2	Axis inhibition protein 2
CKI	Cyclin-dependent kinase inhibitor protein
β-TrCP	Beta-transducin repeat-containing protein
E3	Ubiquitin ligase
FZD	Frizzled gene
LRP5/6	Low-density lipoprotein receptor-related proteins 5 and 6
Myc	A transcription factor belonging to the basic helix–loop–helix leucine zipper (BHLH-LZ) family
Dkk1	A member of the DICKKOPF gene family
MAPK	Mitogen-activated protein kinase
PI3K	Phosphatidylinositol 3-kinases
DIM	3,3′-Diindolylmethane
I3C	Indole-3-carbinol
ML-1	Follicular thyroid cancer cells
CGTH-W-1	Follicular thyroid cancer cells
8505-C	Human papillary thyroid cancer cells
